# Synthesis, Spectroscopy Characterization and Biological Evaluation of La(III), Eu(III) and Gd(III) Complexes with Ampicillin: In Vitro Antimicrobial, Cytotoxic and Antiproliferative Activities and Theoretical Frameworks

**DOI:** 10.3390/molecules31091465

**Published:** 2026-04-28

**Authors:** Diego Boldo, Vasilii Khripun, Kristiane Fanti Del Pino, Juliana Jorge, Luana da Silva Oliveira, Danielle Bogo, Ana Camila Micheletti, Adriana Pereira Duarte, Hernane da Silva Barud, Ariadna Lafourcade Prada, Teofilo Fernando Mazon Cardoso, Gustavo Rocha de Castro, Jesus Rafael Rodríguez Amado, Marco Antonio Utrera Martines

**Affiliations:** 1 Chemistry Institute, Federal University of Mato Grosso do Sul, Campo Grande 79079-900, MS, Brazil; diego.boldo18@gmail.com (D.B.); vasilli.khripun@ufms.br (V.K.); kristianefdp@gmail.com (K.F.D.P.); juliana.jorge@ufms.br (J.J.); luana.s.oliveira@ufms.br (L.d.S.O.); anamicheletti@gmail.com (A.C.M.); adriana.duarte@ufms.br (A.P.D.); 2 Faculty of Pharmacy, Food and Nutrition, Federal University of Mato Grosso do Sul, Campo Grande 79079-900, MS, Brazil; danielle.bogo@ufms.br (D.B.); ariadnalafu1977@gmail.com (A.L.P.); teofilo.cardoso@ufms.br (T.F.M.C.); 3 Laboratory of Biopolymers and Biomaterials, University of Araraquara, Araraquara 14801-340, SP, Brazil; hernane.barud@gmail.com; 4 Institute of Biosciences, São Paulo State University, Botucatu 18618-689, SP, Brazil; gustavo.castro@unesp.br; 5 Faculty of Health Sciences, Federal University of Grande Dourados, Dourados 79825-070, MS, Brazil; amado.jesus@ufam.edu.br

**Keywords:** ampicillin, lanthanides complexes, spectroscopic studies, antimicrobial activity, DFT calculations

## Abstract

This study reports the synthesis, characterization, DFT calculations and in vitro antimicrobial, cytotoxic and antiproliferative evaluation of La(III), Eu(III), and Gd(III) metal complexes with ampicillin. The compounds were characterized by Thermal Gravimetric Analysis (TGA), elemental analysis, ultraviolet–visible spectroscopy (UV–Vis), Fourier-transform infrared spectroscopy (FTIR), and proton nuclear magnetic resonance (^1^H NMR), indicating a 2:1 metal-to-ligand ratio with ampicillin, and likely, a coordination through carbonyl, carboxylic and β-lactam groups, with the general formula [Ln_2_(L)(Cl)_5_(H_2_O)_x_] (Ln = La(III), Eu (III), Gd (III), and x = 2 for La(III), 5 for Eu(III) and Gd(III), L-ampicillin anion). Antimicrobial studies showed activity against ampicillin-resistant *Staphylococcus aureus* (MIC = 15.6 µg·mL^−1^) but no activity against *Escherichia coli*. In cytotoxicity studies, all complexes inhibited B16-F10 (murine melanoma) proliferation, with GI_50_ values around 140 µg·mL^−1^. Against U251 (glioma) cell line, only [Eu_2_(L)(Cl)_5_(H_2_O)_5_] exhibited cytotoxicity activity, GI_50_ = 104 µg·mL^−1^, and notably, [Eu_2_(L)(Cl)_5_(H_2_O)_5_] was active against MCF7 (breast carcinoma) with a GI_50_ = 8.1 µg·mL^−1^. However, all complexes exhibited high cytotoxicity in NIH-3T3 cells (GI_50_ = 0.030–2.90 µg·mL^−1^), indicating limited selectivity between normal and cancer cells. Nevertheless, except for the La complex, most compounds were less cytotoxic than doxorubicin, highlighting the need for further optimization to improve selectivity.

## 1. Introduction

Metallopharmaceuticals are a class of drugs that contain metal ions in their composition, which are often coordinated to an active pharmaceutical compound that acts as a ligand [1]. The coordination process occurs via a Lewis acid–base interaction, in which the metal ions, acting as Lewis acids, accept electron density from ligands, which function as Lewis bases [1,2]. This interaction is facilitated by the partially filled *d* orbitals of transition metals and *f* orbitals of lanthanides, resulting in predominantly ionic coordination complexes [2,3].

Metallopharmaceuticals are promising candidates for drug development, especially considering several studies have already demonstrated an increase in pharmacological activity when drugs are coordinated to metal ions, compared to their free forms. Shamim and co-workers [4] demonstrated that Gemifloxacin, when complexed with heavy metal ions as a bidentate ligand, showed significantly improved antimicrobial activity against various bacterial strains, including *P. mirabilis*, *S. typhi*, *E. coli*, *P. aeruginosa*, *K. pneumoniae*, and *S. flexneri*, while its uncoordinated form did not exhibit such high activity. Similarly, Murcia and co-workers [5] reported that Cr(III) and Co(II) complexes with triazole-based ligands exhibited superior antimicrobial properties relative to the free ligands, with chromium complexes showing MIC_50_ values between 7.8 and 15.6 μg·mL^−1^ against *Candida tropicalis*.

Möhler and co-workers [6] demonstrated that the complexation of β-lactam antibiotics with metal ions (Ag, Cu, and Zn) can enhance their antibacterial efficacy against multidrug-resistant (MDR) strains. In particular, ampicillin/Ag(I) (1:1) complexes showed pronounced activity against *K. pneumoniae* (FICI < 0.48) and *P. aeruginosa* (FICI < 0.47), while penicillin G/Ag(I) (1:0.5) was highly effective against *A. baumannii* (FICI < 0.25). Furthermore, against MRSA strains *(S. aureus)*, penicillin G/Cu(II) (1:1) and ampicillin/Zn(II) (1:10) complexes also exhibited significant synergistic effects, with FICI values below 0.13 and 0.35, respectively.

The study by Kosaristanova and co-workers [7] demonstrated that the synergistic interaction between the iron complex Fe16 and ampicillin (AMP) resulted in a significant enhancement of antibacterial activity against *Staphylococcus aureus*. This effect was evidenced by a marked reduction in individual MIC values, decreasing from 125 μg/mL (Fe16) and 2 μg/mL (AMP) to 31 μg/mL and 0.5 μg/mL, respectively, when used in combination. This interaction was further confirmed by an FICI value of 0.498, classifying the effect as synergistic (≤0.5).

A lot of other examples demonstrating the enhancement of biological activity through metal complexation are known. Suprofen complexes showed increased superoxide dismutase activity compared to the free drug [8]; paracetamol and aspirin complexes displayed variable antibacterial effects [9], and Ru(II) complexes with chloroquine were found to be two to five times more active than the free drug against drug-resistant *Plasmodium falciparum* strains [10].

Lanthanide ions in biological systems are extensively studied not only for their spectroscopic and magnetic properties [11,12,13], but also due to their emerging pharmacological applications. In fact, several lanthanide-based metallodrugs have shown enhanced biological activity. For instance, Eu(III), Sm(III), Gd(III), Dy(III), Ho(III), Er(III), and Yb(III) complexes with 8-hydroxyquinoline-N-oxide and 1,10-phenanthroline exhibited potent antiproliferative effects against cisplatin resistant tumor cells (A549/DDP), with IC_50_ values ranging from 0.025 to 0.097 μM [14]. Complexes of Ce(III) and Sm(III) with 2-acetylpyridine nicotinohydrazone also demonstrated significant in vitro antitumor activity against pancreatic (PATU8988), colorectal (LoVo), and gastric (SGC7901) cancer cell lines, inducing apoptosis as confirmed by Annexin V/PI analysis [15]. Likewise, La(III), Tb(III), Eu(III), and Gd(III) complexes with nalidixic acid exhibited IC_50_ values lower than cisplatin in LoVo cells (breast adenocarcinoma) [16]. Amoxicillin complexes with La(III), Ce(III), Sm(III), and Y(III) also showed enhanced antimicrobial and antiparasitic activity, along with effects on tumor cell lines [17]. Moreover, La(III), Nd(III), Sm(III), Eu(III), Gd(III), and Tb(III) complexes with usnic acid presented antitumor activity against MCF-7 cells and demonstrated anti-leishmanial properties [18,19].

Furthermore, different studies indicate that lanthanide ion complexes often exhibit higher selectivity and activity against tumor cells when coordinated to anticancer drugs [20,21,22]. The cytotoxic effects of Ln(III) complexes, including gadolinium(III) texaphyrin, are primarily attributed to reactive oxygen species (ROS)-mediated DNA damage, leading to the activation of p53-dependent apoptotic signaling pathways [23]. Recent studies have shown that metal-based anticancer agents, including Pt, Ru, Au, Cu, Ir, and Os complexes, can exert significant in vivo antitumor effects. While many compounds display potent in vitro cytotoxicity, several have been demonstrated to reduce tumor growth and improve survival in animal models, often with improved tolerability compared to conventional platinum drugs. These findings highlight the potential of structurally diverse metallodrugs for further clinical development [24].

Lanthanide ions, particularly in oxidation state +3 (Ln(III)), can closely mimic biologically essential metal ions like calcium (Ca(II)), and to some extent, other M(II) and M(III)cations [25]. This resemblance arises mainly from similarities in ionic radii and coordination tendencies, allowing lanthanides to substitute calcium in certain biological systems and protein structures. In specific coordination environments, lanthanide ions may exhibit stronger binding than calcium, depending on the structural characteristics of the binding site and the ligand composition. This preference is associated with their classification as hard Lewis acids, which tend to coordinate preferentially with hard donor atoms, particularly oxygen-containing ligands, rather than softer donors such as nitrogen, phosphorus, or sulfur [26]. This unique ability renders lanthanides highly valuable in molecular biology and bioimaging. Nonetheless, their strong binding affinity also raises important safety concerns regarding their potential to disrupt biological pathways if used without proper regulation [27,28].

The gradual decrease in ionic radius across the lanthanide series, a phenomenon known as the lanthanide contraction [26], also contributes to their chemical versatility. This trend affects how lanthanides form complexes and how closely they can emulate the roles of naturally occurring metal ions [26]. This property is not only fundamental to their biological behavior but also underpins their application in metal ion separation, sensing technologies, and purification processes in complex systems [25,28].

Therefore, considering the distinctive properties of lanthanides and previous reports indicating that drug metal complexation can influence biological activity, this work is devoted to synthesis, characterization, theoretical insights, and biological evaluation of metal-based compounds, with a focus on the in vitro antimicrobial, cytotoxic, and antiproliferative activities of La(III), Eu(III), and Gd(III) ion complexes with ampicillin.

## 2. Results and Discussion

### 2.1. Synthesis of the Complexes LaL, EuL and GdL

**Scheme 1:**
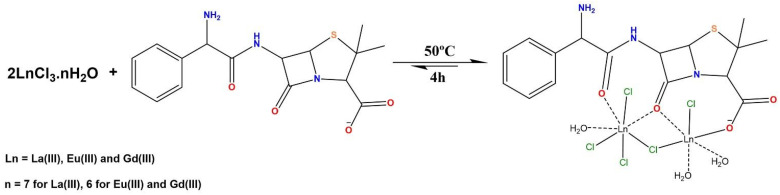
The synthesis process of the complexes [La_2_(L)(Cl)_5_(H_2_O)_2_], [Eu_2_(L)(Cl)_5_(H_2_O)_4_] and [Gd_2_(L)(Cl)_5_(H_2_O)_5_].

The complexation reaction of La(III), Eu(III), and Gd(III) ions with the ligand ampicillin (in the form of sodium salt) is evidenced by the formation of yellow-colored precipitates (Figure S1), resulting from the addition of an aqueous–ethanolic solution of lanthanide chlorides to sodium ampicillin, as shown in Scheme 1 [3,4,18].

Furthermore, the complexes exhibit similar physical properties and solubility. All complexes were insoluble in polar protic and nonpolar solvents, but only soluble in polar aprotic and coordinating solvent (DMSO), since the lanthanides used share the same oxidation states, Ln(III), similar ionic radius (La = 1.06 Å, Eu = 0.95 Å, and Gd = 0.94 Å), and the *f* orbitals have minimal involvement in the complexation reactions, which are predominantly ionic [3,26].

### 2.2. Thermal and Elemental Analysis

**Figure 1:**
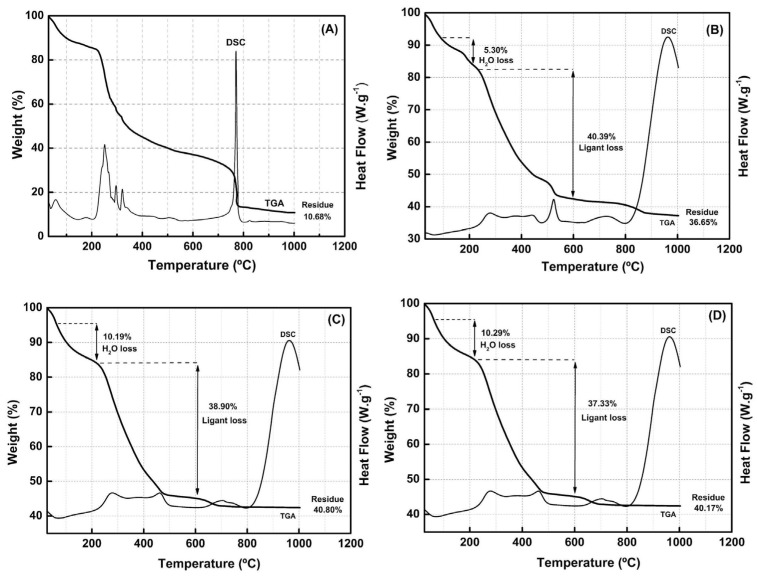
Thermoanalytical curves TGA and DSC: (**A**) ampicillin L, ampicillin sodium salt, NaL; (**B**) [La_2_(L)(Cl)_5_(H2O)_2_]; (**C**) [Eu_2_(L)(Cl)_5_(H_2_O)_5_]; (**D**) Gd_2_(L)(Cl)_5_(H_2_O)_5_].

The thermal analysis of ampicillin sodium salt (Figure 1A, Table 1) demonstrates that the first mass loss is characteristic of hydration water molecules (30.60–190.76 °C), followed by the decomposition of the ligand (190.76–950 °C), with the formation of sodium oxide at the end (950 °C) [3].

The thermal analysis of the complexes (Figure 1B–D, Table 1) shows similar mass loss profiles as follows: (a) the first step refers to the loss of hydration water molecules (25–100 °C) (1); (b) the second exothermic step, occurring at a temperature close to 300 °C, can be related to the pyrolysis of organic fragment and the onset of lanthanide–ligand bond breakage, (2); (c) formation of La_2_O_3_, Eu_2_O_3_, and Gd_2_O_3_ oxides (800 °C) [3,17,18] (2).

[Ln_2_(L)(Cl)_5_(H_2_O)_x_](s) → [Ln_2_(L)(Cl)_5_] + xH_2_O(g) (1)

[Ln_2_(L)(Cl)_5_] → L_(g)_ + 5Cl^−^_(g)_ + Ln_2_O_3(s)_ (2)

Furthermore, based on the DSC curves of the synthesized complexes (Figure S2), it was possible to confirm that these complexes have coordination water molecules, since all curves showed broad endothermic peaks, starting at temperatures above 100 °C and extending to temperatures greater than 150 °C with high dehydration enthalpy values [29], as shown in Table S1, consistent with the presence of coordination water [30].

The analysis of the TGA and DSC curves of the complexes LaL, EuL, and GdL synthesized allows us to conclude that the lanthanide–ligand ratio is 2:1, which is also confirmed by elemental analysis and complexometric titration (Table S2). Thus, the molecular formulas for these complexes are LaL = [La_2_(L)(Cl)_5_(H_2_O)_2_], EuL = [Eu_2_(L)(Cl)_5_(H_2_O)_5_], and GdL = [Gd_2_(L)(Cl)_5_(H_2_O)_5_], and in addition, all of these complexes are amorphous (Figure S3).

### 2.3. Spectroscopy Characterization

**Figure 2:**
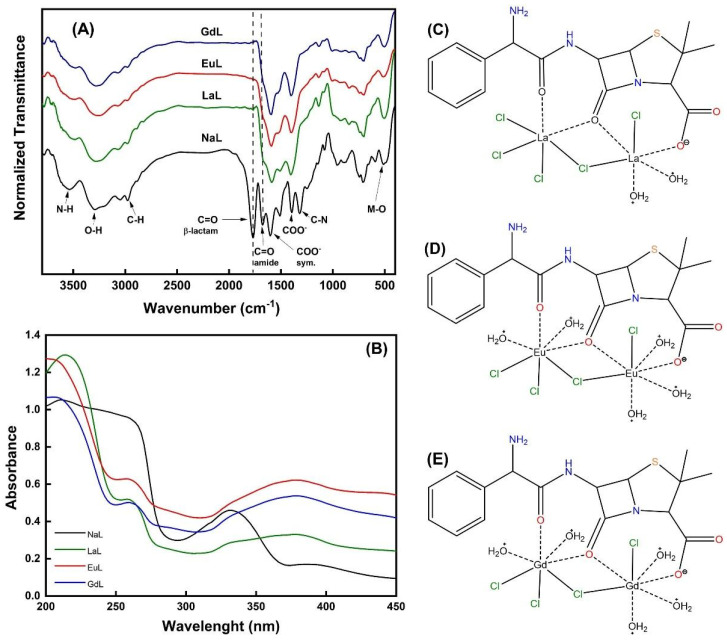
(**A**) FTIR spectra, (**B**) electronic spectra for ligand (as sodium salt, NaL) and La, Eu and Gd complexes and the proposed structures for them. LaL = [La_2_(L)(Cl)_5_(H_2_O)_2_] (**C**); EuL = [Eu_2_(L)(Cl)_5_(H_2_O)_5_] (**D**); GdL = [Gd_2_(L)(Cl)_5_(H_2_O)_5_] (**E**).

To describe the coordination of the ampicillin to the metal centers, IR spectra (Figure 2A, Table 2) and the UV-Vis (Figure 2B, Table S3) of the complexes were recorded. In the UV-Vis spectra, the π → π* transition related to the aromatic ring is found at 266 nm for the free ligand, and for the complexes of La(III), Eu(III), and Gd(III), it is shifted to 267 nm [3,17]. The n → π* transition, characteristic of the carbonyl groups of the β-lactam ring, is observed at 342 nm for the free ligand and for the complexes La(III), Eu(III), and Gd(III) as well, at 384 nm, 381 nm, and 381 nm, respectively [3].

The UV–Vis results obtained in this work are consistent with those reported in the literature for β-lactam–metal coordination systems. In particular, Refat and co-workers [3] reported similar bathochromic shifts in the absorption bands to approximately 362 nm in analogous lanthanide complexes (La(III), Ce(III), Sm(III), and Y(III)) with amoxicillin, which were attributed to coordination at the carbonyl site of the β-lactam ring, confirming that metal–ligand coordination significantly affects the electronic transitions of the ligand. This behavior can be rationalized based on Pearson’s hard and soft acids and bases theory, according to which lanthanide(III) ions, classified as hard acids, preferentially coordinate to hard bases such as the oxygen atoms of carbonyl groups, which are highly electronegative donor atoms [1,2,3,17].

The IR spectra of the complexes (Figure 2A, Table 3) corroborate the coordination behavior observed in the UV–Vis data, providing complementary evidence for metal–ligand interactions. It is well-established that β-lactam antibiotics, such as ampicillin, can coordinate to transition metal and lanthanide ions through amino, carbonyl, and carboxylate groups, including the β-lactam ring [3,17,31,32,33].

In line with these data, the IR spectra obtained in this study indicate that ampicillin coordinates to the lanthanide centers through multiple donor sites, including, carbonyl (amide and β-lactam) and carboxylate groups, as proposed in Figure 2C–E. This assignment is supported by the following observations:

(1) The bands at 3500 and 3300 cm^−1^, attributed to ν(N–H) and ν(O–H) stretching vibrations, respectively, show reduced intensity and broadening in the complexes, indicating the involvement of the amino group and the presence of coordinated or lattice water molecules [3,17,33]. 

(2) The bands at 3050 and 2970 cm^−1^, corresponding to ν(Csp^2^–H) and ν(Csp^3^–H), as well as the band at ~1508 cm^−1^ associated with aromatic ν(C=C), remain essentially unchanged, indicating that the aromatic ring does not participate directly in coordination [3,17].

(3) The characteristic bands at 1774 cm^−1^ (ν(C=O)_β-lactam_) and 1670 cm^−1^ (ν(C=O)_amide_), clearly observed in the free ligand, are absent or significantly reduced in the complexes. This behavior is consistent with coordination through carbonyl groups, as widely reported in the literature presented in Table 2, and supports their direct involvement in metal binding. 

(4) The bands around 1600 cm^−1^, assigned to asymmetric and symmetric stretching of the carboxylate group ν(COO^−^), exhibit slight shifts to lower wavenumbers in the complexes, indicating coordination through carboxylate moiety [3,17,31,32,33,34].

(5) New bands appearing near 500 cm^−1^, attributed to ν(M–O) vibrations, further confirm the formation of metal–oxygen bonds involving carbonyl and carboxylate groups, and are absent in the free ligand [3,17,31].

Therefore, the combined spectroscopic data demonstrate that ampicillin acts as a multidentate ligand, coordinating to the lanthanide ions through amino, carbonyl, and carboxylate groups, in full agreement with previously reported coordination modes for β-lactam-based systems. In agreement with this behavior, Refat and co-workers [3] demonstrated that lanthanide complexes of amoxicillin (La(III), Ce(III), Sm(III), and Y(III)) involve coordination through the carbonyl group of the β-lactam ring, leading to weakening or disappearance of the ν(C=O) band upon complexation. Similarly, Al-Khodir and Refat [17] reported that ampicillin complexes with Fe(III), Pd(II), and Au(III) exhibit coordination through ν(C=O)amide, ν(C=O)β-lactam, and ν(N–H)imino groups, with corresponding bands showing reduced intensity or disappearance. In addition, El-Gamel [31] observed that bands at 1761–1763 cm^−1^, assigned to the β-lactam carbonyl group, disappear upon coordination, confirming its direct involvement in chelation.

Further supporting this coordination mode, Abbas and co-works [32] reported that ampicillin-derived Schiff base complexes with Co(II), Ni(II), Cu(II), Eu(III), and Gd(III) coordinate through the amide nitrogen, β-lactam carbonyl, and amino groups. More recently, Justyna Frymark and co-workers [35] demonstrated that complexes of ampicillin with alkaline earth, transition metals, and lanthanides (including Nd(III), Eu(III), and Tb(III)) involve coordination through the carboxylate, amide carbonyl, β-lactam ring, and amino groups, depending on the system composition.

**Figure 3:**
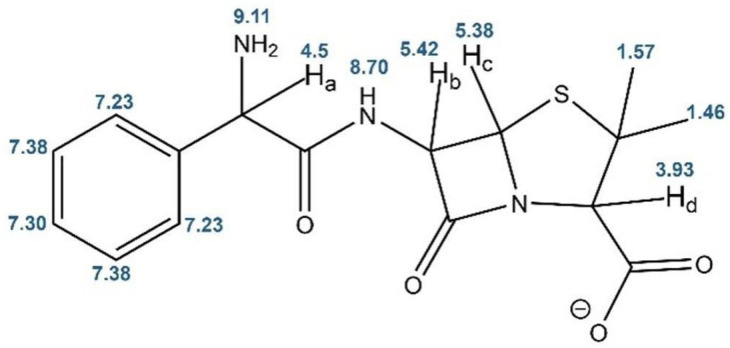
Molecular structure of the ligand with chemical shifts (δ in ppm) attributed to each hydrogen atom.

The presence of Ln(III) ions in ^1^H nuclear magnetic resonance (NMR) spectra can be characterized by relaxation, broadening, and sometimes the shifting of signals to a chemical shift (δ) different from those for the free ligand [17,36]. Certain Ln(III) ions possess unpaired *4f* electrons, leading to an anisotropic effect [3,36]. The H^1^ NMR spectrum of the ligand (Figure S4) and its assignments are shown in Figure 3 and Table 4. The signals of the benzene hydrogens appear at δ = 7.38, 7.30, and 7.28 ppm, respectively [17].

The signal for the hydrogen in the N-H group appears as a singlet at δ = 8.70 ppm, while the signal for the two hydrogens in the NH_2_ group appears as a doublet at δ = 9.11 ppm [17]. The signals for hydrogens H_a_, H_b_, H_c_, and H_d_ appeared at δ = 4.55, 5.42, 5.38, and 3.93 ppm, respectively [17].

H_b_ and H_c_ experience the anisotropic effect caused by the carbonyl group present in the β-lactam ring; however, H_b_ is affected by the nitrogen in the amide bond, and H_c_ is influenced by the electronegativity of the sulfur atom, resulting in slight differences in chemical shifts between them. Hydrogen H_a_ also experiences an anisotropic effect, which is attributed to the carbonyl group of the amide bond and the electrons of the aromatic ring, resulting in higher δ ppm values compared to hydrogens H_b_ and H_c_ [17].

The spectrum of the complex LaL is shown in Figure S4B, and the assignments are given in (Table 5). The signals of the aromatic ring hydrogens are at δ = 7.48, 7.41, and 7.33 ppm, with broadened, “distorted” profiles shifted to higher ppm values, indicating the proximity of the lanthanum atom to this group. Furthermore, the signals for the hydrogens in the NH and NH_2_ groups shift to lower values (δ = 8.66 and 8.26 ppm, respectively), which can be attributed to the intramolecular hydrogen bond (H_2_N–H^+^) [3,17]. Additionally, the signals of H_b_, H_c_, and H_a_ hydrogens shift to higher δ ppm values (δ = 7.1, 6.87, and 4.70 ppm, respectively).

The signal for H_d_ also shifts to higher δ ppm values (δ = 4.54 ppm), and the signals for the methyl group hydrogens split into six signals ranging from δ = 1.5 to 1.1 ppm. These effects are attributed to the second lanthanum atom coordinated to the β-lactam carbonyl oxygen and the oxygen of the carboxylate group, reinforcing the hypotheses of coordination of two lanthanum ions to ampicillin by the carboxyl and β-lactam carbonyl groups [3,17].

Spectroscopic data elucidates the corresponding coordination sites of the metal ions and the structures of the complexes. In the case of the [La_2_(L)(Cl)_5_(H_2_O)_2_] complex, both La(III) ions have a coordination number (CN) of 6. The first site, located near the carbonyl region, involves coordination with three chloride ions in addition to the carbonyl groups from the β-lactam ring and the amide moiety. The second site is bound to the carboxylate fragment and is completed with two chloride ions and two water molecules.

In contrast, although Eu(III) and Gd(III) ions typically exhibit high coordination numbers (CN = 8, 9, or even 10) [37], the EuL and GdL complexes display asymmetric coordination numbers. At the first site, located near the carbonyl groups, a CN = 7 was proposed, suggesting that structural factors such as steric hindrance may limit the number of ligands around the metal center, even for lanthanides that generally favor higher coordination [37,38].

This decrease in CN can be attributed to the steric hindrance imposed by bulky ligand groups. Ligands such as cyclooctatetraenyl derivatives or those containing amide and formamidines moieties may physically obstruct the approach of additional ligands, favoring more stable configurations with CN = 7 [37,38].

Edelmann and co-workers [37] reported that lanthanide complexes with cyclooctatetraenyl (COT) ligands containing bulky trimethylsilyl substituents, despite their tendency to form well-defined sandwich-type structures, exhibit a smaller number of coordinated solvent molecules (e.g., THF) as ligand steric demand increases, leading to a decrease in coordination number from 8 to 7. Additionally, the authors highlighted those complexes derived from the parent COT^2−^ dianion often present significant limitations in terms of solubility and crystallinity. These observations reinforce that increasing ligand steric hindrance not only influences the immediate coordination environment but also impacts macroscopic properties such as crystal formation, further evidencing the challenges associated with obtaining well-ordered single crystals in sterically hindered lanthanide systems.

Similarly, Le Borgne and co-workers [39] demonstrated that peripheral substitution on ONO tridentate ligands, specifically, ethyl, isopropyl, and benzyl, affects the formation and geometry of lanthanide complexes. They observed that, as the steric bulk of the side chains increases (ethyl < isopropyl < benzyl), the metal–ligand coordination affinity decreases, consequently lowering the coordination number. In addition, the authors report that single crystals obtained for dicarboxamidopyridine-based systems are often fragile and highly solvated, requiring the use of very slow ether diffusion techniques in concentrated solutions to enable their formation. In this context, the isolation of suitable crystals for structural analysis may occur selectively and is, in some cases, described by the authors as a rare event. These difficulties demonstrate that solid-state organization in these systems is not a trivial step, but a process highly dependent on factors such as steric hindrances of ligands, interaction with solvent molecules, and the stability of the species formed in solution.

M.A and co-workers [40] also emphasized that the spatial constraints imposed by formamidinato ligands, especially those containing 2,6-diisopropylphenyl substituents, reduce the number of coordinated solvent molecules (e.g., THF), giving a value of around 7. This behavior is further enhanced by intermolecular interactions between units, confirming that steric hindrance, in combination with crystal packing effects, plays a decisive role in the observed coordination architecture. Furthermore, the authors report the difficulty in obtaining single crystals of the 5b complex, attributing this difficulty to its low solubility and its crystal habit in the form of very fine needles.

Therefore, the coordination numbers for Eu(III) and Gd(III) complexes with ampicillin (particularly the CN = 7 at the site containing carbonyl groups adjacent to aromatic rings) are consistent with those previously reported for lanthanide complexes with bulky ligands. The presence of sterically demanding groups near donor atoms imposes spatial constraints that limit coordination numbers.

As reported by Edelmann [37], Le Borgne [39] and M.A and co-workers [40], both steric hindrance and molecular packing directly influence the geometry and saturation of coordination, justifying the observed structural asymmetry. These factors reinforce that the decrease in coordination number does not arise from instability or coordination deficiency but rather represents a structural adaptation of the system in response to the ligand’s bulk and its local steric environment.

In addition to these structural considerations, it is important to highlight that such steric and coordination features also have direct implications for crystallization behavior. In systems such as the one investigated in this work, the combination of bulky ligand environments, flexible coordination geometries, and possible solvent interactions tends to hinder the formation of well-ordered crystalline lattices, making the isolation of suitable single crystals particularly challenging. This behavior is consistent with the literature discussed above, in which lanthanide complexes bearing sterically demanding ligands frequently exhibit low crystallinity, high solvation, or the formation of microcrystalline or poorly diffracting solids. Therefore, the difficulty in obtaining single crystals for the present complexes should not be interpreted as a limitation of the synthetic approach, but rather as a direct consequence of the intrinsic structural and supramolecular characteristics of these systems.

### 2.4. Theoretical Calculations

The synthesized lanthanide complexes with ampicillin are amorphous, so it was not possible to grow single crystals suitable for X-ray structural analysis. The structures shown in Figure 2C are proposed based on spectral data. However, ampicillin has wide coordination capabilities and, thus, can form different bonds with metal centers due to other donor atoms and functional groups. For example, the imide fragment or sulfur atom of ampicillin is capable of coordinating to the metal center. In addition, the carboxyl group quite easily forms chelate complexes with lanthanides. To confirm the proposed structures, a quantum–chemical calculation was performed for the lanthanum complex with ampicillin.

For the calculation, a functional Cam-B3LYP [41] and SDD basis set [41,42,43,44,45,46,47] were used, which is quite popular for describing the structures of lanthanides and IR spectra [48,49,50]. The calculated structure of the ampicillin complex with lanthanum, the obtained coordinates of the atoms of the complex, and the calculated IR spectrum are given in the Supplementary Materials (Figures S5 and S6, Table S5). Coordination numbers for both lanthanum atoms are 6, which is quite typical for this metal.

**Figure 4:**
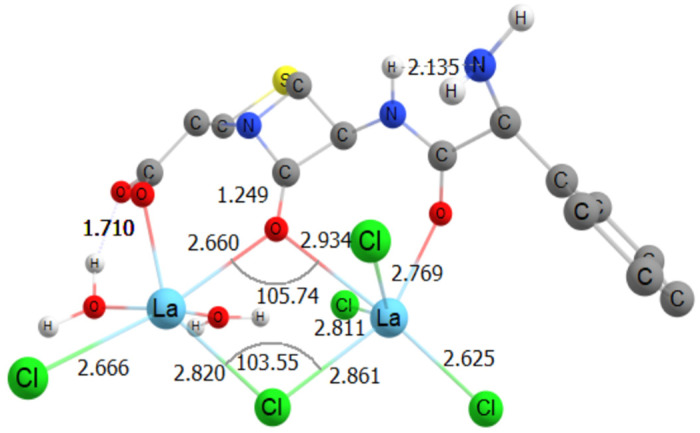
A fragment of the structure of the LaL complex with selected angles and bond lengths.

First, it is worth noting that in the different starting geometry, the carboxyl group acted as a chelate; also, there was a bond between the imide nitrogen and lanthanum, and all chloride ions occupied the terminal position. The resulting calculated IR spectrum (Figure S5) is quite close to the experimental one (Figure 2A), which indicates a well-optimized geometry in the DFT calculation. A fragment of the structure of the lanthanum complex with optimized geometry with selected angles and bond lengths is shown in Figure 4.

The steric strain of the ligand chain does not allow a stable bond to be formed with the imide nitrogen atom and lanthanum, and the chelate coordination of the carboxyl group also seems to be unstable. Instead, the carbonyl group, due to the oxygen atom as well as one of the chlorides, occupies bridging positions between two lanthanum ions. Although terminal coordination of chloride is more favorable, known structures contain a bridging position of chloride ion between two lanthanide ions [51,52,53]. It should be noted that the terminal coordination of the carboxyl group is stabilized by the presence of a hydrogen bond with one of the coordinated water molecules (Figure 4). In addition, the proton of the imide group is placed quite close to the nitrogen of the amino group (intermolecular bond length is about 2.1 Å), which allows one to assume the possibility of forming a hydrogen bond in solution. As a result, the signal of the imide proton as well as protons of amino groups are shifted in the NMR spectrum.

The structural characteristics of the known complexes [51,52] are quite close to our calculations. Thus, the valence angle La-Cl-La is 103.50, and the lengths of the bridge chloride–lanthanum bonds are ca 2.8 Å. It is likely the case that this bridge position of the chloride as well as carbonyl group of the ampicillin increased the biological activity of the synthesized complexes. This is due to the fact that in biological systems, chloride and carbonyl can move to the terminal position at one of the lanthanum atoms, thus opening a coordination vacancy at the other, increasing its reactivity.

### 2.5. Antimicrobial Assay

**Figure 5:**
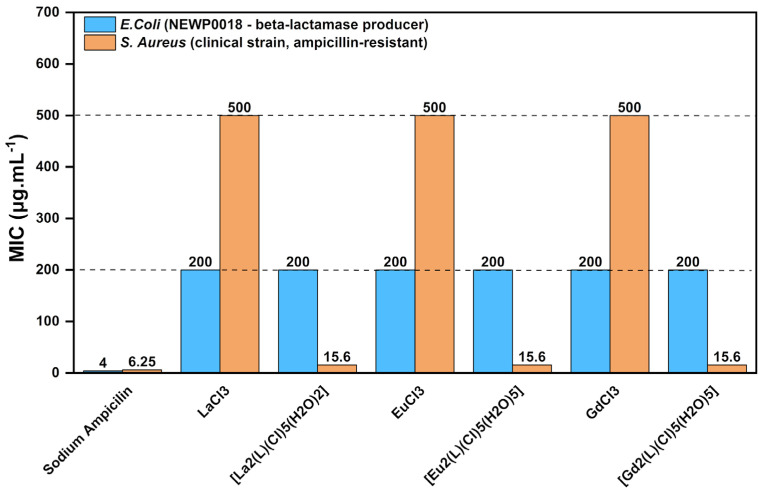
Antimicrobial activity in vitro of the ligand (NaL), the lanthanides salts (LnCl_3_, Ln = La(III), Eu(III) and Gd(III), and the synthesized complexes, LaL = [La_2_(L)(Cl)_5_(H_2_O)_2_]_,_ EuL = [Eu_2_(L)(Cl)_5_(H_2_O)_5_]Cl_2_ and GdL = [Gd_2_(L)(Cl)_5_(H_2_O)_5_], against the following strains: *Staphylococcus aureus* (clinical strain, ampicillin-resistant, Gram-positive) and *Escherichia coli* (NEWP0018–Gram-negative, commercial strain, beta-lactamase producer).

In this work, the antimicrobial activity of the complexes, [La_2_(L)(Cl)_5_(H_2_O)_2_], [Eu_2_(L)(Cl)_5_(H_2_O)_5_] and [Gd_2_(L)(Cl)_5_(H_2_O)_5_], were tested against *S. aureus* and *E. coli* (both resistant to ampicillin) and compared with the neutral ligand (ampicillin salt sodium) and its salt forms (LaCl_3_, EuCl_3_ and GdCl_3_) (Figure 5). The complexes demonstrated activity against the Gram-positive strain (*S. aureus*); the minimum inhibitory concentration (MIC) results were 15.6 µg·mL^−1^ for all complexes and 18.56 μM, 16.94 μM, and 16.75 μM for [La_2_(L)(Cl)_5_(H_2_O)_2_], [Eu_2_(L)(Cl)_5_(H_2_O)_5_], and [Gd_2_(L)(Cl)_5_(H_2_O)_5,_ respectively, and 6,25 µg·mL^−1^ (16.82 μM) for free ampicillin. However, both complexes exhibited no significant activity against the Gram-negative bacterium *Escherichia coli*, with MIC values ≥ 200 µg·mL^−1^, well above the threshold commonly considered indicative of low antimicrobial activity [54].

The lower antibacterial activity observed for the complexes [La_2_(L)(Cl)_5_(H_2_O)_2_], [Eu_2_(L)(Cl)_5_(H_2_O)_5_], and [Gd_2_(L)(Cl)_5_(H_2_O)_5_] compared to the free ligand can be rationalized based on structural and mechanistic considerations. The antibacterial activity of ampicillin, as a β-lactam antibiotic, is strictly dependent on the integrity and reactivity of the β-lactam ring, particularly the carbonyl group, which is essential for the acylation of penicillin-binding proteins (PBPs) [55]. In this context, coordination of Ln(III) centers through the carbonyl oxygen of the β-lactam moiety is expected due to hard acid–hard base interactions [1,21], leading to a redistribution of electron density and a decrease in the electrophilicity of this group. Such electronic modulation can significantly impair the nucleophilic attack required for PBP inhibition, thereby reducing antibacterial efficacy.

Furthermore, the coordination of ampicillin to lanthanide ions may limit the availability of the free drug under biological conditions, which could contribute to the reduced antibacterial activity observed. Although no direct stability studies under physiological conditions were performed, this hypothesis is consistent with the literature reports indicating that ligand coordination and the resulting electronic and steric modifications can alter or even diminish antimicrobial activity when key functional groups are involved in metal binding [56,57]. In particular, recent work on the attachment of metal-containing moieties to β-lactam antibiotics has shown that modifications of the β-lactam scaffold through metal coordination can significantly change the chemical behavior and properties of the antibiotic framework, reinforcing the notion that preservation of the β-lactam pharmacophore is critical for biological activity [58].

Moreover, the efficacy of the bioactive complexes demonstrated that they may represent an innovative strategy in the battle against bacterial resistance because antibiotic resistance, particularly in strains of *Staphylococcus aureus*, represents a major challenge in modern medicine. This bacterium is recognized as one of the primary pathogens responsible for nosocomial infections, accounting for a substantial proportion of hospital-acquired infections worldwide [59]. Its resistance is often exacerbated by the production of beta-lactamases, enzymes capable of inactivating beta-lactam antibiotics, such as ampicillin, thereby complicating infection management and treatment outcomes [59,60]. In this context, the development of new bioactive compounds, such as the complexes presented herein, exhibiting a minimum inhibitory concentration (MIC) of 15.6 µg·mL^−1^ against resistant strains of *S. aureus*, is of interest.

Finally, the introduction of new antimicrobial agents, such as the bioactive complexes discussed herein, is critical not only for treating resistant infections but also for addressing the escalating global threat of antibiotic resistance. A comprehensive review published by Okeke co-workers [61] reported that in 2019, an estimated 4.95 million deaths were associated with drug-resistant bacterial infections, of which approximately 1.27 million were directly attributable to antimicrobial resistance (AMR). The study warns that, without urgent global action, this number could rise significantly by 2050, compromising not only routine medical procedures but also global food security and sustainable development goals [61].

### 2.6. Evaluation, Cytotoxicity and Antiproliferative Activity of the Synthesized Complexes in Cell Lines B16-F10, U251, MCF7

Cytotoxic activity is determined based on GI_50_ values, which represent the concentrations capable of inhibiting cell growth by 50%, expressed in µg ml^−1^ and µM [62,63]. Samples are considered active against the tested cell lines when GI_50_ values are <250 μg·mL^−1^, inactive when GI_50_ values are ≥250 μg·mL^−1^, and selective when the selectivity index (SI) is greater than or equal to 2.0 [63].

The cytotoxic activity of the synthesized complexes, lanthanide salts, and the ligand are presented in Table 5 for the B16-F10 (murine melanoma), U251 (glioma), MCF7 (breast carcinoma) cell lines, and the NIH-3T3 (murine fibroblast) cell line. The ligand in its free form exhibited no activity against the tested cell lines. For the B16-F10 cell line, the lanthanide salts showed no activity, whereas the complexes exhibited activity with GI_50_ values of 140 µg·mL^−1^ (LaL), 135 µg·mL^−1^ (EuL), and 140 µg·mL^−1^ (GdL). In the U251 cell line, neither the ligand nor the lanthanide salts, and GdL and LaL complex, displayed activity. However, the EuL complex did show activity with a GI_50_ value of 104 µg·mL^−1^. Against the MCF-7 cell line, the lanthanide salts, the free ligand, and the GdL complex exhibited no significant cytotoxic activity. In contrast, the LaL and EuL complexes demonstrated cytotoxic potential, with GI_50_ values of 50 µg·mL^−1^ and 8.1 µg·mL^−1^, respectively. For the NIH-3T3 cell line, all tested compounds presented GI_50_ values below 50 µg·mL^−1^, ranging from 0.06 µg·mL^−1^ to 5.9 µg·mL^−1^. Notably, the LaL complex showed the highest cytotoxicity, with a GI_50_ of 0.06 µg·mL^−1^, indicating greater activity than both the free ligand (GI_50_ = 2.6 µg·mL^−1^) and the lanthanide salt (GI_50_ = 3.5 µg·mL^−1^).

**Figure 6:**
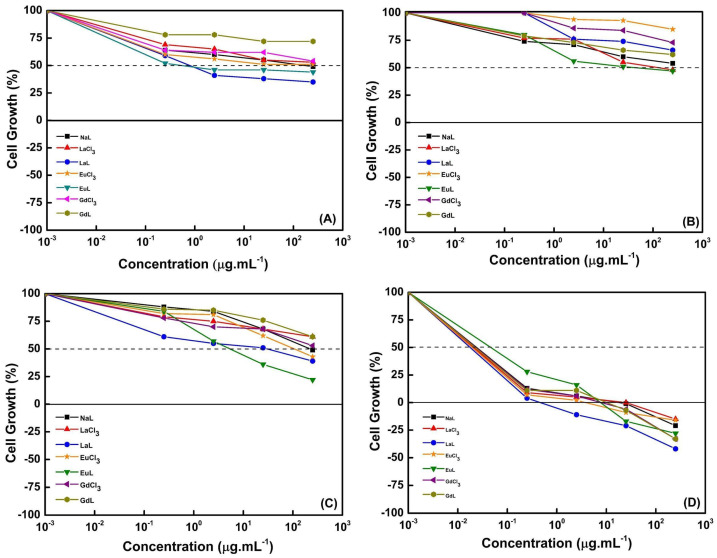
Antiproliferative activity in vitro of the free ligand (NaL), the lanthanide chlorides (LnCl_3_, Ln = La(III), Eu(III) and Gd(III)), and the synthesized complexes, LaL = [La_2_(L)(Cl)_5_(H_2_O)_2_]_,_ EuL = [Eu_2_(L)(Cl)_5_(H_2_O)_5_] and GdL = [Gd_2_(L)(Cl)_5_(H_2_O)_5_] against the following cell lines: (**A**) B16-F10, (**B**) U251, (**C**) MCF7 and (**D**) NIH-3T3 exposed to different concentrations (0.25, 2.5, 25 and 250 μg·mL^−1^) for 48 h.

The antiproliferative activity curves for the B16-F10, U251, MCF7, and NIH-3T3 cell lines are shown in Figure 6A–D, respectively, at different concentrations tested for each of the Ln complexes and chlorides and the free ligand. The points where the growth curves intersect the line estimating the calculated GI_50_ value indicate the cytostatic effect, while the cytocidal effect corresponds to the points below zero on the curve [63,64].

The complexes LaL and EuL exhibit a cytostatic effect on the B16-F10 cell line at concentrations close to 2.5 µg·mL^−1^ (Figure 6A), specifically 2.48 µg·mL^−1^ (LaL) and 2.53 µg·mL^−1^ (EuL), however, without demonstrating a cytocidal effect. The EuL complex exhibits a cytostatic effect on the U251 cell line at concentrations around 25 µg·mL^−1^ (25.50 µg mL^−1^) (Figure 6B). Additionally, lanthanum chloride also shows a cytostatic effect, but at higher concentrations, namely 250 µg·mL^−1^, neither group of samples shows a cytocidal effect. Concerning the MCF7 cell line (Figure 6C), a cytostatic effect is observed only for the EuL and LaL complexes at concentrations close to 25 µg·mL^−1^ (25.22 µg·mL^−1^ and 25.04 µg·mL^−1^, respectively). Europium chloride and the GdL complex exhibit a cytostatic effect only at higher concentrations, 250 µg·mL^−1^. However, a cytocidal effect was not observed in any of the cases.

From a pharmacological perspective, ampicillin, a β-lactam antibiotic, is primarily designed to target bacterial cell wall biosynthesis and is not expected to display intrinsic antitumor activity. This is consistent with the limited cytotoxicity observed for sodium ampicillin in the present study. Nevertheless, previous reports have demonstrated that structural modifications of ampicillin can significantly alter its biological profile. For instance, Ferraz and co-workers [65] described ampicillin-based ionic liquids exhibiting marked antiproliferative activity against multiple human cancer cell lines, including T47D, PC3, HepG2, MG63, and RKO, with IC_50_ values in the nanomolar range (5–42 nM).

However, it is worth noting that the Eu(III) (EuL) complex exhibits a cytostatic effect in all cell lines evaluated, occurring at approximately 2.5 µg·mL^−1^ for the B16-F10 cell line and at higher concentrations (around 25 µg·mL^−1^) for the U251 and MCF7 cell lines. For the nontumor tissue cell line, NIH-3T3 (murine fibroblast), all samples tested showed a cytostatic effect at concentrations as low as 0.25 µg·mL^−1^, progressing to a cytocidal effect at approximately 2.5 µg·mL^−1^. This behavior indicates a pronounced cytotoxic response in nontumor cells; although the complexes exhibit lower cytotoxicity than the control drug, doxorubicin, the results indicate low selectivity.

A plausible explanation for the observed cytotoxicity of the complexes may be related to their binuclear structures, namely [La_2_(L)(Cl)_5_(H_2_O)_2_], [Eu_2_(L)(Cl)_5_(H_2_O)_5_], and [Gd_2_(L)(Cl)_5_(H_2_O)_5_], as well as to the well-established behavior of lanthanide coordination compounds in biological environments. In particular, Ln(III) ions are known to act as strong Lewis acids according to the hard and soft acids and bases (HSAB) theory, and may exhibit a high affinity for negatively charged donor atoms, especially oxygen-containing groups such as carboxylates, phosphates, phenolates, and carbonyl functionalities commonly found in biomolecules [1,21]. Accordingly, Ln(III) ions may interact with DNA through multiple modes, including electrostatic interactions with the phosphate backbone, groove binding, and, in some cases, intercalation, which can lead to structural perturbations and may contribute to cytotoxic effects [21,22,23,24].

Furthermore, the binuclear nature of these complexes may play a role in enhancing their biological activity. As reported by Alexander and co-workers [66], binuclear lanthanide architectures can create preorganized binding pockets between the metal centers, enabling simultaneous interactions with anionic substrates such as chloride ions, phosphate groups, and even double-stranded DNA. This cooperative binding mode can increase affinity compared to mononuclear analogs, which are less effective in coordinating such species in aqueous environments.

In addition, the presence of chloride ligands in the coordination sphere may further influence biological activity, as these complexes are inevitably exposed to highly aqueous intracellular environments, where the cytoplasm is predominantly composed of water [67] and contains a wide range of competing ligands, such as chloride ions, phosphates, and biomacromolecules. In aqueous solution, lanthanide–chloride interactions are known to be highly dynamic, with chloride ions capable of occupying both inner and outer-sphere coordination positions and undergoing rapid exchange with solvent molecules [68]. Therefore, this lability may facilitate ligand substitution processes under physiological conditions, potentially leading to the formation of more reactive species [66].

This type of coordination behavior has been reported by Aramesh-Boroujeni and co-workers [69] who demonstrated that the structure of the complex [Tb(Me_2_Phen)_2_Cl_3_(H_2_O)] is intrinsically correlated with its biological performance, as the DNA cleavage efficiency of lanthanide complexes is highly dependent on the coordination environment and ligand framework. In that system, the coordination environment provided by phenanthroline ligands and chloride ions may enable the Tb(III) ion (4f^8^ configuration) to participate in DNA cleavage mechanisms, including direct activation of phosphodiester bonds as well as oxidative pathways, in addition to promoting groove-binding interactions with DNA.

Regarding the evaluated cytotoxic effects on murine fibroblast cells (NIH-3T3) being extremely high, this can be mitigated using nanoparticle systems. The encapsulation of these complexes in nanoparticles allows us to control targeted release, reducing exposure of non-cancerous cells to the therapeutic agent while enhancing selectivity for tumor cells [70,71,72].

Systems such as polymeric nanoparticles or liposomes have demonstrated potential in improving selective drug delivery [72], as evidenced by Torchilin [71], who discussed the advantages of encapsulating therapeutic agents in nanoparticles for both passive and active targeting. Furthermore, the functionalization of nanoparticles with ligands that recognize receptors overexpressed in cancer cells can further increase the specificity of the treatment [72].

Another benefit of using nanoparticles is the improvement in the pharmacokinetics and biodistribution of metal complexes, particularly through the enhanced permeability and retention (EPR) effect, resulting in greater accumulation of the therapeutic agent in tumor tissues [73].

## 3. Materials and Methods

### 3.1. Materials

Sodium ampicillin, Europium (III) chloride hexahydrate (EuCl_3_·6H_2_O), Lanthanum (III) chloride heptahydrate (LaCl_3_·7H_2_O) and Gadolinium (III) chloride hexahydrate (GdCl_3_·6H_2_O) were purchased from Sigma-Aldrich. (Cajamar, SP, Brazil) Solvents (PA grade) were used without any further purification. Cell lines MCF7 (ATCC^®^–HTB-22™, breast carcinoma) and U251 (glioma) were acquired by Roswell Park Memorial Institute and cell lines B16-F10 (ATCC^®^-CRL-6475™, murine melanoma) and NIH-3T3 (ATCC^®^-CRL-1658™, murine fibroblast) (UNICAMP, BR). All culture media for antimicrobial assays were purchased from Sigma-Aldrich. *Escherichia coli* (NEWP0018) strain was acquired from Newprov Company. *Staphylococcus aureus* (clinical strain, resistant to ampicillin) was provided by the Center of Clinical Analysis of the University Hospital, Universidade Federal de Mato Grosso do Sul (Campo Grande, Brazil). Ethanol (CH_3_CH_2_OH, EMSURE^®^, ≥99.8%, Merck, Darmstadt, Germany).

### 3.2. Synthesis of the La(III), Eu(III) and Gd(III) Complexes Coordinated to Ampicillin

The complexes were synthesized based on modified pathways [3,17]. The metal-to-ligand molar ratio was 1:3. An amount of 3 mmol (1.11 g) of sodium ampicillin (NaL) was dissolved in 5 mL of ethanol. An amount of 1 mmol of lanthanide chloride (EuCl_3_·6H_2_O, LaCl_3_·7H_2_O, GdCl_3_·6H_2_O) was dissolved in 5 mL of ethanol and was slowly added to the sodium ampicillin solution. The reaction was kept under constant stirring (500 rpm) for 4 h at 50 °C. The precipitate formed was filtrated in vacuum using a 0.45 µm filter. The precipitate was washed three times with ethanol and dried at room temperature in a desiccator.

**NaL**: FTIR (Kbr, cm^−1^) 3550 (N-H), 3300 (O-H), 3050 (C-H), 1774 (C=O)_β-lactam_, 1671 (C=O)_amide_, 1604 (COO−)_asymmetric_, 1317 (COO−)_symmetric_, 1317 (C-N), 1508 (C=C), 1706 (C-O), 495 (M-O). NMR ^1^H (DMSO d_6_) δ (ppm), 8.70 (NH), 9.11 (NH_2_), 7.38, 7.30, 7.2 (aromatic ring), 4.5 (Ha), 5.42 (Hb), 5.38 (Hc), 3.93 (Hd), 3.45 (H_2_O), 1.56, 1.47 (CH_3_)

**[La_2_(L)(Cl)_5_(H_2_O)_2_]**: FTIR (Kbr, cm^−1^) 3550 (N-H), 3288 (O-H), 3048 (C-H), absent (C=O)_β-lactam_, absent (C=O)_amide_, 1585 (COO-)_asymmetric_, 1407 (COO-)_symmetric_, absent (C-N), 1498 (C=C), 1132 (C-O), 501 (M-O). NMR ^1^H (DMSO d6) δ (ppm), 8.66 (NH), 8.26 (NH_2_), 7.48, 7.41, 7.33 (aromatic ring), 4.70 (Ha), 7.10 (Hb), 6.87 (Hc), 4.54 (Hd), 3.64 (H_2_O), 1.50–1.10 (CH3)

**[Eu_2_(L)(Cl)_5_(H_2_O)_5_]**: FTIR (Kbr, cm^−1^) 3550 (N-H), 3266 (O-H), 3047 (C-H), absent (C=O)_β-lactam_, absent (C=O)_amide_, 1590 (COO-)_asymmetric_, 1408 (COO-)_symmetric_, absent (C-N), 1504 (C=C), 1130 (C-O), 501 (M-O).

**[Gd_2_(L)(Cl)_5_(H_2_O)_5_]**: FTIR (Kbr, cm^−1^) 3550 (N-H), 3286 (O-H), 3048 (C-H), absent (C=O)_β-lactam_, absent (C=O)_amide_, 1591 (COO-)_asymmetric_, 1405 (COO-)_symmetric_, absent (C-N), 1503 (C=C), 1130 (C-O), 501 (M-O).

### 3.3. Thermogravimetric Analysis

The curves were obtained using the Q50 model from TA Instruments (TA Instruments, New Castle, DE, USA), under an O_2_ atmosphere. The heating rate was 10 °C.min^−1^ and temperature ranged from 25 °C to 1000 °C in the Pt crucible.

### 3.4. Infrared Spectroscopy

The spectra of the ligand and of the complexes synthesized were registered in a wavenumber range from 4000 to 400 cm^−1^, using KBr pellets with resolution 4 cm^−1^, on a Nicolet iS5 spectrometer from Thermo Scientific (Thermo Fisher Scientific, Waltham, MA, USA), at the Laboratório de Tecnologia Farmacêutica (LTF), Universidade Federal de Mato Grosso do Sul, Campo Grande, Brazil.

### 3.5. Electronic Absorption Spectroscopy

The electronic molecular absorption spectra in the ultraviolet and visible (UV-Vis) regions were registered on a Lambda 60S–PerkinElmer spectrophotometer (PerkinElmer, Waltham, MA, USA). The solid-state spectroscopic analyses were performed using the diffuse reflectance technique. The spectra registered were used after transforming the percentage of reflectance into absorbance [log10(1/reflectance)] in the spectrophotometer with an integrating sphere.

### 3.6. Elemental Analysis (CHN)

The analysis was performed using a PerkinElmer 2400 series (PerkinElmer, Waltham, MA, USA) II Flash Combustion Analyzer from the LCC-CNRS laboratory in Toulouse, Toulouse, France.

### 3.7. X-Ray Diffraction

The analysis was performed using a Bruker XRD D2 Phaser (Bruker, Billerica, MA, USA), with a monochromatic Cu X-Ray source at a wavelength of 1.54184 Å, operating at 30 kV and 10 mA. The scan was performed over a 2θ range from 8° to 80°, with a 0.002° step, a 10 s interval between steps, and a rotation speed of 15 RPM.

### 3.8. Proton Nuclear Magnetic Resonance H^1^ Spectroscopy

The H^1^ spectra were recorded using a Bruker DPX 300 NMR (Bruker, Billerica, MA, USA) spectrometer operating at a frequency of 600 MHz, using DMSO-d^6^ as the solvent with the reference being the residual solvent signal.

### 3.9. Antimicrobial Assays: Determination of the Minimum Inhibitory Concentration (CMI)

The antibacterial activity was performed by the broth microdilution method [74]. The 96-well plates were prepared by adding 100 μL of Mueller–Hinton broth to each well. Then, 100 μL of a solution with concentration of 4 mg·mL^−1^ in dimethyl sulfoxide (DMSO) was added to the first well. Subsequently, 100 μL from this well was transferred to the second one, and successive 1:2 dilutions were performed to achieve final concentrations ranging from 2000 μg·mL^−1^ to 0.98 μg·mL^−1^, with a final volume of 100 μL in each well. For ampicillin, the final concentration in the wells varied between 200 μg·mL^−1^ and 1.56 μg·mL^−1^. The bacterial inoculum consisted of a 24 h culture of each bacterial species [*Staphylococcus aureus* (clinical strain, ampicillin-resistant, Gram-positive) and *Escherichia coli* (NEWP0018–Gram-negative, commercial strain, beta-lactamase producer)] grown on Mueller–Hinton agar and diluted in sterile saline solution (0.9%) to a concentration of 10^8^ CFU.mL^−1^. This approximately concentrated solution was diluted 1/10 in sterile saline, and 5 μL was added to each well. All tests were performed in triplicate, and the plates were incubated at 36 °C for 18 h. After this period, 20 μL of an aqueous (0.5%) triphenyl tetrazolium chloride (TTC) solution was added to each well, and the plates were incubated again at 36 °C for 2 h. In the wells where bacterial growth occurred, a color change from colorless to red was observed. The MIC was defined as the lowest concentration of each substance where no color change was detected.

### 3.10. Cytotoxicity Assays of LaL, EuL, and GdL Complexes by the Sulforrodamina B (SRB) Method

The colorimetric method used in adherent cells was the sulforrodamina B (SRB) assay, as described by Papazisis and collaborators [75]. After obtaining the appropriate number of cells, they were detached from the maintenance flasks (bottles) by trypsin-EDTA action (initial procedure performed as previously described for cell passaging) and transferred to a conical tube with complete medium (three times the volume of trypsin) and centrifuged for 4 min at 1000 rpm.

The pellet was resuspended in 2 mL of culture medium, and an aliquot of this suspension was taken and diluted in trypan blue (1:4) to highlight non-viable cells, which were excluded from the count. At this stage, a manual counter (Neubauer chamber) was used to obtain a cell suspension, sufficient to inoculate 5000 cells for the U251 cell line and 7500 cells for the other lines in 100 µL of medium, per well in a 96-well test plate. After cell counting, the T0 (Time Zero) plate and the test plate were prepared. To the T0 plate, a triplicate of the cell suspension for each cell line and a triplicate of the culture medium were added. The T0 plate was then incubated, and after 24 h, the staining procedure (described below) was performed by adding sulforrodamina B (SRB), a dye that binds to cell. The T0 plate serves as the baseline for the experiment, and after reading it, it is possible to determine the density of viable cells when the samples were added to the test plate. For the test plate containing the cell suspensions, after the initial 24 h incubation, the test samples were added in triplicate at concentrations of 0.25, 2.5, 25, and 250 μg·mL^−1^, and the plate was incubated for another 48 h. The test plate contained a blank for each test sample concentration, a negative control (cells plus 100 µL of medium), and a positive control (doxorubicin at 0.025, 0.25, 2.5, and 25 μg·mL^−1^). After the 48 h incubation, the staining procedure with SRB was performed. The staining procedure was carried out as follows: The supernatant was aspirated from the wells, leaving only the cells. Then, 100 µL of cold 20% trichloroacetic acid (TCA) was added to each well to fix the viable cells, and the plates were left to rest for 30 min at 4 °C, protected from light. The supernatant was discarded, and the plates were washed with running water. After drying, 50 µL of 0.1% SRB (Sigma, St. Louis, MO, USA) diluted in 1% acetic acid was added. The plates were again left for 30 min at room temperature, protected from light. The plates were washed five times with 1% acetic acid to remove excess of unbound dye. Subsequently, 100 µL of Trizma Base buffer ((10 mM, pH 10.5) (Cajamar, SP, Brazil)) was added to each dry well to solubilize the dye bound to the proteins of the fixed cells. The plates were then shaken for 15 min, followed by absorbance reading at 540 nm using a Molecular Devices SpectraMax 190^®^ (Molecular Devices, San Jose, CA, USA) microplate reader. With the assistance of ORIGIN^®^ 6.0 software, the concentration required to inhibit 50% of cell proliferation, referred to as GI_50_ (Growth Inhibition) [75], was determined using non-linear regression analysis.

### 3.11. Theorical Calculations

The calculations were performed using the Gaussian 16 program (revision A.03), using computational resources provided by Superintendência de Tecnologia da Informação da Universidade de São Paulo [76]. All structures were fully optimized; the energy minimum was checked by analytical calculation of the frequencies of normal vibrations. Geometry optimizations and calculations of the frequencies of normal vibrations were performed at the DFT level using the Cam-B3LYP functional [41]. The SDD basis set was chosen for the lanthanum atom [41,42,43,44,45,46,47]. For the remaining elements, the 6-31G basis set was used [77,78,79,80]. The Chemcraft program (Version 1.8 (build 809b)) was used to visualize the optimized geometries and normal vibrations [81].

## 4. Conclusions

This study reports the synthesis of three novel La(III), Eu(III), and Gd(III) complexes with ampicillin in a 2:1 metal-to-ligand ratio. The structures were characterized by spectroscopic techniques, indicating that coordination occurs mainly through the carbonyl and carboxylate groups. DFT calculations further suggest binuclear arrangements in which the metal centers are bridged by carbonyl groups and chloride ions, contributing to the structural organization of the complexes.

All synthesized complexes exhibited antimicrobial activity against ampicillin-resistant *Staphylococcus aureus* (MIC = 15.6 µg·mL^−1^); however, their activity was lower than that of the free ligand. This behavior can be attributed to the involvement of the β-lactam carbonyl group in metal coordination, which is a key pharmacophoric site required for the interaction with penicillin-binding proteins (PBPs). These results reinforce that preservation of the β-lactam core is essential for maintaining antibacterial efficacy, and that coordination at this site may be detrimental to activity.

In cytotoxicity assays, all complexes showed inhibitory effects against B16-F10 (murine melanoma) cells, with GI_50_ values around 140 µg·mL^−1^. The europium complex also demonstrated activity against U251 (glioma) and MCF7 (breast carcinoma) cell lines, with a more pronounced effect in the latter (GI_50_ = 8.1 µg·mL^−1^). However, the observed cytotoxicity toward NIH-3T3 cells indicates low selectivity between tumor and nontumor cells, limiting their potential as therapeutic agents in their current form. This behavior may be associated with structural features such as the binuclear arrangement and the presence of labile chloride ligands, which can influence interactions in biological environments.

Overall, although coordination did not lead to improved antimicrobial or selective cytotoxic activity, this study provides valuable insights into the structure–activity relationships of lanthanide–β-lactam systems. The results highlight the importance of preserving key pharmacophoric groups and suggest that future strategies should focus on alternative coordination modes or structural modifications to improve selectivity and biological performance.

## Figures and Tables

**Table 1:** Calculated and experimental values (%) of total mass losses, loss of the ligand and water for the complexes from TGA analysis, and calculated and experimental percentages of carbon, hydrogen, nitrogen, and lanthanides, where LaL = [La_2_(L)(Cl)_5_(H_2_O)_2_], EuL = [Eu_2_(L)(Cl)_5_(H_2_O)_5_] and GdL = [Gd_2_(L)(Cl)_5_(H_2_O)_5_].

	Total Loss (%)		Ligand Loss (%)		H_2_O Loss (%)		%C		%H		%N		%Ln	
Compound	Calc.	Exp.	Calc.	Exp.	Calc.	Exp.	Calc.	Exp.	Calc.	Exp.	Calc.	Exp.	Calc.	Exp.
LaL	61.19	63.37	41.50	40.39	4.30	5.30	22.89	23.53	2.65	2.90	5.01	5.22	33.09	33.42
EuL	61.74	59.27	37.88	38.90	9.78	10.19	20.90	21.74	3.07	3.50	4.57	5.10	33.05	33.58
GdL	61.04	59.83	37.45	37.33	9.67	10.29	20.66	21.97	3.03	3.80	4.52	5.05	33.81	34.67

Calc. = calculated and Exp. = experimental data; residue LaL (La_2_O_3_), EuL (Eu_2_O_3_) and GdL (Gd_2_O_3_).

**Table 2:** β-lactam complexes with transitions and lanthanides metals found in the literature. ACT—amoxicillin; Amp and Hamp—ampicillin; AA—ampicillin derivative.

Coordination Formulas	Coordination Sites	References
[La(ACT)(H2O)_3_]⋅(NO_3_)_2_⋅H_2_O	C=O_β-lactam_; −N_imino_; N_amine_	[3]
[Ce(ACT−NH_4_)(H_2_O)_3_]⋅(NO_3_)_3_⋅H_2_O	C=O_β-lactam_; −N_imino_; N_amine_	[3]
[Sm(ACT)(H_2_O)_3_]⋅(NO_3_)_2_⋅H_2_O	C=O_β-lactam_; −N_imino_; N_amine_	[3]
[Y(ACT−NH_4_)(H_2_O)_3_]⋅(NO_3_)_3_⋅H_2_O	C=O_β-lactam_; −N_imino_; N_amine_	[3]
[Fe_2_(amp)(Cl)_5_(H_2_O)_3_].3H_2_O	C=O_β-lactam_; C=O_amide_; N_amide_; COO^−^	[17]
[Pd(amp)_2_].2H_2_O	C=O_β-lactam_; COO^−^	[17]
[Au_2_(amp)(Cl)_4_].Cl.2H_2_O	C=O_β-lactam_; C=O_amide_; N_amide_; COO^−^	[17]
[Ce(III)(Hamp)(Cl)_2_].(H_2_O)	C=O_β-lactam_; −N_imino_; N_amide_; COO^−^	[31]
[Nd(III)(Hamp)(H_2_O)_2_].NO_3_	C=O_β-lactam_; −N_imino_; N_amide_; COO^−^	[31]
[Th(IV)(Hamp)(H_2_O)_2_].(NO_3_)_2_	C=O_β-lactam_; −N_imino_; N_amide_; COO^−^	[31]
[Eu(AA)(H_2_O)Cl]Cl.6H_2_O	C=O_β-lactam_; N_amide_	[32]
[Gd(AA)(H_2_O)(NO_3_)]NO_3_.2H_2_O	C=O_β-lactam_; N_amide_	[32]

**Table 3:** Assignment of the infrared bands of the ligand, NaL, and the lanthanides complexes, where asy = asymmetric stretching, sym. = symmetric stretching, L = C_16_H_18_N_3_SO−, LaL = [La_2_(L)(Cl)_5_(H_2_O)_2_], EuL = [Eu_2_(L)(Cl)_5_(H_2_O)_5_], and GdL = [Gd_2_(L)(Cl)_5_(H_2_O)_5_].

	Assignments (cm^−1^)										
	υ(N-H)	υ(O-H)	υ(C-H)	υ(C=O) _β-lactam_	υ(C=O) _amide_	υ_asy._ (COO^−^)	υ_sym._ (COO^−^)	υ_sym._ (C-N)	υ(C=C)	υ(C-O)	υ(M-O)
Compound											
NaL	3550	3300	3050	1774	1671	1604	1317	1317	1508	1706	495
LaL	3550	3288	3048	-	-	1585	1407	-	1498	1132	501
EuL	3550	3266	3047	-	-	1590	1408	-	1504	1130	501
GdL	3550	3286	3048	-	-	1591	1405	-	1503	1130	501

**Table 4:** Assignments of the chemical shifts in H_1_ (ppm) for the ligand and the complex. LaL = [La_2_(L)(Cl)_5_(H_2_O)_2_].

	Chemical Shift (δ) in ^1^H NMR Spectra (ppm).									
Compound	NH	NH_2_	Aromatic Ring	Ha	Hb	Hc	Hd	H_2_O	CH_3_	Ref.
NaL	8.70	9.11	7.38, 7.30, 7.23	4.50	5.42	5.38	3.93	3.45	1.56, 1.47	[3,17]
LaL	8.66	8.26	7.48, 7.41, 7.33	4.70	7.10	6.87	4.54	3.64	1.50–1.10	

**Table 5:** GI_50_ values of in vitro cytotoxicity for the ligand, lanthanide chlorides and La(III), Eu(III) and Gd(III) complexes with ampicillin on murine melanoma cells (B16-F10), glioma cells (U251), breast carcinoma cells (MCF-7) and normal cells, murine fibroblast (NIH-3T3), after 48 h of treatment. GI_50_ ≥ 250 μg·mL^−1^ values indicate no activity.

	GI_50_ Cell Lines							
	B16-F10		U251		MCF-7		NIH-3T3	
Compound	μg·mL^−1^	μM	μg·mL^−1^	μM	μg·mL^−1^	μM	μg·mL^−1^	μM
NaL	≥250	-	≥250	-	≥250	-	2.60	7.00
LaCl_3_.7H_2_O	≥250	-	≥250	-	≥250	-	3.50	9.42
[La_2_(L)(Cl)_5_(H_2_O)_2_]	140.0	166.6	≥250	-	50.0	59.5	0.03	0.036
EuCl_3._6H_2_O	≥250	-	≥250	-	≥250	-	1.70	4.64
[Eu_2_(L)(Cl)_5_(H_2_O)_5_]	135.0	146.6	104	113.0	8.10	8.80	1.40	1.52
GdCl_3._6H_2_O	≥250	-	≥250	-	≥250	-	2.90	7.80
[Gd_2_(L)(Cl)_5_(H_2_O)_5_]	140.0	150.3	≥250	-	≥250	-	2.50	2.68
Doxorubicin	0.025	0.05	0.20	0.40	0.15	0.30	0.10	0.20

## Data Availability

The original contributions presented in this study are included in the article/Supplementary Materials. Further inquiries can be directed to the corresponding author.

## Supplementary Materials

The following supporting information can be downloaded at: https://www.mdpi.com/article/10.3390/molecules31091465/s1 [82,83,84], Figure S1: Physical appearance of the synthesized complexes with their respective colors: (A) ampicillin coordinated with lanthanum (LaL); (B) ampicillin coordinated with europium (EuL); (C) ampicillin coordinated with gadolinium (GdL); Figure S2: DSC analysis of complexes: (A) LaL = [La_2_(L)(Cl)_5_(H_2_O)_2_], (B) EuL = [Eu_2_(L)(Cl)_5_(H_2_O)_5_] and (C) GdL = [Gd_2_(L)(Cl)_5_(H_2_O)_5_]; Table S1: Dehydration enthalpy values of the lanthanide complexes; Table S2: Volumes of 0.01 mol·L^−1^ EDTA used in the complexometric titrations and the corresponding percentages of lanthanide in the respective samples; Figure S3: Diffractogram of the synthesized complexes, LaL, EuL, and GdL; Table S3: Assignments of the bands and their respective electronic transitions for the ligand (NaL) and the corresponding complexes [La_2_(L)(Cl)_5_(H_2_O)_2_], [Eu_2_(L)(Cl)_5_(H_2_O)_5_], and [Gd_2_(L)(Cl)_5_(H_2_O)_5_], where L = sodium ampicillin salt; Figure S4: ^1^H NMR spectra in DMSO-d_6_: (A) of the ligand; (B) [La_2_(L)(Cl)_5_(H_2_O)_2_]; Table S4: Electrical conductivity measurements of the ligand and the synthesized complexes at a concentration of 10^−3^ M in DMSO at 26 °C at 0, 24, 48, and 72 h; Table S5: Coordination of atoms in La_2_(L)(Cl)_5_(H_2_O)_2_ complex; Figure S5: The structure of calculated La_2_(L)(Cl)_5_(H_2_O)_2_ complex; Figure S6: Calculated IR spectrum of the La_2_(L)(Cl)_5_(H_2_O)_2_ complex.

## Abbreviations

The following abbreviations are used in this manuscript:

DMSO	Dimethyl sulfoxide
DMSO-d^6^	Deuterated dimethyl sulfoxide
DSC	Differential scanning calorimetry
GI_50_	Concentration required to inhibit 50% of cell growth

## References

[1] Sadeer N.B., Varghese R., Ramamoorthy S., Zengin G. (2024). Coordination and bioinorganic chemistry at the heart of metallodrugs: Knowledge gaps and future directions. Inorg. Chim. Acta.

[2] Bünzli J.C.G. (2014). Lanthanide coordination chemistry: From old concepts to coordination polymers. J. Coord. Chem..

[3] Refat M.S., Al-Maydama H.M., Al-Azab F.M., Amin R.R., Jamil Y.M. (2014). Synthesis, thermal and spectroscopic behaviors of metal–drug complexes: La(III), Ce(III), Sm(III) and Y(III) amoxicillin trihydrate antibiotic drug complexes. Spectrochim. Acta A.

[4] Shamim S., Gul S., Rauf A., Rashid U., Khan A., Amin R., Akhtar F. (2022). Gemifloxacin-transition metal complexes as therapeutic candidates: Antimicrobial, antifungal, anti-enzymatic, and docking studies of newly synthesized complexes. Heliyon.

[5] Murcia R.A., Leal S.M., Roa M.V., Nagles E., Muñoz-Castro A., Hurtado J.J. (2018). Development of Antibacterial and Antifungal Triazole Chromium(III) and Cobalt(II) Complexes: Synthesis and Biological Activity Evaluations. Molecules.

[6] Möhler J.S., Kolmar T., Synnatschke K., Hergert M., Wilson L.A., Ramu S., Elliott A.G., Blaskovich M.A., Sidjabat H.E., Paterson D.L. (2017). Enhancement of antibiotic-activity through complexation with metal ions—Combined ITC, NMR, enzymatic and biological studies. J. Inorg. Biochem..

[7] Kosaristanova L., Rihacek M., Sucha F., Milosavljevic V., Svec P., Dorazilova J., Vojtova L., Antal P., Kopel P., Patocka Z. (2023). Synergistic antibacterial action of the iron complex and ampicillin against *Staphylococcus aureus*. BMC Microbiol..

[8] Underhill A.E., Bougourd S.A., Flugge M.L., Gale S.E., Gomm P.S. (1993). Metal complexes of anti-inflammatory drugs. Part VIII: Suprofen complex of copper(II). J. Inorg. Biochem..

[9] Lawal A., Obaleye J.A. (2007). Synthesis, characterization and antibacterial activity of aspirin and paracetamol metal complexes. Biokemistri.

[10] Sánchez-Delgado R.A., Navarro M., Pérez H., Urbina J.A. (1996). Toward a novel metal-based chemotherapy against tropical diseases. 2. Synthesis and antimalarial activity in vitro and in vivo of new ruthenium- and rhodium-chloroquine complexes. J. Med. Chem..

[11] Bünzli J.C., Choppin G.R. (1989). Lanthanide Probes in Life, Chemical and Earth Sciences.

[12] Zhu Z., Guo M., Li X.L., Tang J. (2019). Molecular magnetism of lanthanide: Advances and perspectives. Coord. Chem. Rev..

[13] Rhee M.J., Sudnick D.R., Arkle V.K., Horrocks W.D. (1981). Lanthanide ion luminescence probes. Characterization of metal ion binding sites and intermetal energy transfer distance measurements in calcium-binding proteins. 1. Parvalbumin. Biochemistry.

[14] Yang Y., Zhou Z., Wei Z.-Z., Qin Q.-P., Yang L., Liang H. (2021). High anticancer activity and apoptosis- and autophagy-inducing properties of novel lanthanide(III) complexes bearing 8-hydroxyquinoline-N-oxide and 1,10-phenanthroline. Dalton Trans..

[15] Xu Z.Q., Mao X.J., Jia L., Xu J., Zhu T.F., Cai H.X., Bie H.Y., Chen R.H., Ma T.L. (2015). Synthesis, characterization and anticancer activities of two lanthanide (III) complexes with a nicotinohydrazone ligand. J. Mol. Struct..

[16] Maciuca A.-M., Munteanu A.-C., Mihaila M., Badea M., Olar R., Nitulescu G.M., Munteanu C.V.A., Uivarosi V. (2022). A Study on Repositioning Nalidixic Acid via Lanthanide Complexation: Synthesis, Characterization, Cytotoxicity and DNA/Protein Binding Studies. Pharmaceuticals.

[17] Al-Khodir F.A.I., Refat M.S. (2015). Spectroscopic Elaboration and Structural Characterizations of New Fe(III), Pd(II), and Au(III) Ampicillin Complexes: Metal-Antibiotic Ligational Behaviors. J. Pharm. Innov..

[18] Nunes D.M., Pessatto L.R., Mungo D., Oliveira R.J., de Campos Pinto L.M., da Costa Iemma M.R., Duarte A.P. (2020). New complexes of usnate with lanthanides ions: La(III), Nd(III), Tb(III), Gd(III), synthesis, characterization, and investigation of cytotoxic properties in MCF-7 cells. Inorg. Chim. Acta.

[19] da Silva F., Rizk Y.S., das Neves A.R., Lourenço E.M.G., Ferreira A.M.T., Monteiro M.M., de Arruda C.C.P. (2023). Antileishmanial activity, toxicity and mechanism of action of complexes of sodium usnate with lanthanide ions: Eu(III), Sm(III), Gd(III), Nd(III), La(III) and Tb(III). Int. J. Mol. Sci..

[20] Kostova I., Manolov I., Nicolova I., Konstantinov S., Karaivanova M. (2001). New lanthanide complexes of 4-methyl-7-hydroxycoumarin and their pharmacological activity. Eur. J. Med. Chem..

[21] Costanzo M., Bianco S., Fik-Jaskółka M., Roviello G.N. (2026). Recent Advances in Lanthanide Complexes in Biological Systems: Coordination Principles and Interactions with Biomolecules. Int. J. Mol. Sci..

[22] Teo R.D., Termini J., Gray H.B. (2016). Lanthanides: Applications in cancer diagnosis and therapy: Miniperspective. J. Med. Chem..

[23] Young S.W., Qing F., Harriman A., Sessler J.L., Dow W.C., Mody T.D., Hemmi G.W., Hao Y., Miller R.A. (1996). Gadolinium(III) texaphyrin: A tumor selective radiation sensitizer that is detectable by MRI. Proc. Natl. Acad. Sci. USA.

[24] Paprocka R., Wiese-Szadkowska M., Janciauskiene S., Kosmalski T., Kulik M., Helmin-Basa A. (2022). Latest developments in metal complexes as anticancer agents. Coord. Chem. Rev..

[25] Nikolova V., Kircheva N., Dobrev S., Angelova S., Dudev T. (2023). Lanthanides as Calcium Mimetic Species in Calcium-Signaling/Buffering Proteins: The Effect of Lanthanide Type on the Ca^2+^/Ln^3+^ Competition. Int. J. Mol. Sci..

[26] Cotton F.A., Wilkinson G. (1988). Advanced Inorganic Chemistry.

[27] Amoroso A., Pope S. (2015). Using lanthanide ions in molecular bioimaging. Chem. Soc. Rev..

[28] Wooten D. (2024). A plasmid containing the human metallothionein-II gene selectively distinguishes trivalent lanthanum from several divalent heavy metal cations during monoclonal antibody-assisted agarose gel electrophoresis. Toxicol. Ind. Health.

[29] Zapała L., Kosińska M., Woźnicka E., Byczyński Ł., Zapała W. (2016). Synthesis, spectral and thermal study of La(III), Nd(III), Sm(III), Eu(III), Gd(III) and Tb(III) complexes with mefenamic acid. J. Therm. Anal. Calorim..

[30] Wang Z.L., Niu C.J., Liu Z.H., Ni J.Z. (1996). Synthesis and thermo-decomposition mechanism of rare earth complexes with phenylacetic acid. Thermochim. Acta.

[31] El-Gamel N.E. (2010). Metal chelates of ampicillin versus amoxicillin: Synthesis, structural investigation, and biological studies. J. Coord. Chem..

[32] Abbas A.M., Fisal S.R., Orabi A.S. (2020). Novel β-lactam antibiotic derivative and its complexes: DFT, frontier energy levels, DNA interaction, docking, physicochemical and antimicrobial properties. J. Mol. Struct..

[33] Regupathy S., Nair M.S.S. (2010). Studies on Cu(II) ternary complexes involving an aminopenicillin drug and imidazole containing ligands. Spectrochim. Acta A Mol. Biomol. Spectrosc..

[34] Deacon G.B., Phillips R.J. (1980). Relationships between the carbon-oxygen stretching frequencies of carboxylato complexes and the type of carboxylate coordination. Coord. Chem. Rev..

[35] Frymark J., Zabiszak M., Grajewski J., Tylkowski B., Jastrzab R. (2025). Insights into Complex Compounds of Ampicillin: Potentiometric and Spectroscopic Studies. Int. J. Mol. Sci..

[36] Parker D., Suturina E.A., Kuprov I., Chilton N.F. (2020). How the ligand field in lanthanide coordination complexes determines magnetic susceptibility anisotropy, paramagnetic NMR shift, and relaxation behavior. Acc. Chem. Res..

[37] Edelmann A., Lorenz V., Hrib C., Hilfert L., Blaurock S., Edelmann F. (2013). Steric effects in lanthanide sandwich complexes containing bulky cyclooctatetraenyl ligands. Organometallics.

[38] Cotton S.A. (2005). Establishing coordination numbers for the lanthanoids in simple complexes. Comptes Rendus Chim..

[39] Le Borgne T., Bénech J.-M., Floquet S., Bernardinelli G., Aliprandini C., Bettens P., Piguet C. (2003). Monometallic lanthanide complexes with tridentate 2,6-dicarboxamidopyridine ligands. Influence of peripheral substitutions on steric congestion and antenna effect. Dalton Trans..

[40] Ying-Zhao M.A., Pushkarevsky Y., Sukhikh N., Galashov T., Makarov A., Roesky P., Konchenko S. (2018). Steric influence and intermolecular interactions of formamidinate ligands in lanthanide (Sm, Yb) arylchalcogenolate complexes. Eur. J. Inorg. Chem..

[41] Yanai T., Tew D.P., Handy N.C. (2004). A new hybrid exchange-correlation functional using the Coulomb-attenuating method (CAM-B3LYP). Chem. Phys. Lett..

[42] Igel-Mann G., Stoll H., Preuss H. (1988). Pseudopotentials for main group elements (IIIA through VIIA). Mol. Phys..

[43] Andrae D., Häusermann U., Dolg M., Stoll H., Preuss H. (1990). Energy-adjusted ab initio pseudopotentials for the 2nd and 3rd row transition-elements. Theor. Chim. Acta.

[44] Kaupp M., Schleyer P.V.R., Stoll H., Preuss H. (1991). Pseudopotential approaches to CA, SR, and BA hydrides. Why are some alkaline-earth MX_2_ compounds bent?. J. Chem. Phys..

[45] Küchle W., Dolg M., Stoll H., Preuss H. (1994). Energy-adjusted pseudopotentials for the actinides. Parameter sets and test calculations for thorium and thorium molecules. J. Chem. Phys..

[46] Leininger T., Nicklass A., Stoll H., Dolg M., Schwerdtfeger P. (1996). The accuracy of the pseudopotential approximation. II. A comparison of various core sizes for indium pseudopotentials in calculations for spectroscopic constants of InH, InF, and InCI. J. Chem. Phys..

[47] Cao X., Dolg M. (2002). Segmented contraction scheme for small-core lanthanide pseudopotential basis sets. J. Mol. Struct. (Theochem).

[48] Palafox M.A., Todorov L.T., Belskaya N.P., Álvarez-Conde J., Díaz-García D., Gómez-Ruiz S., Kostova I.P. (2025). The Spectroscopic Characterization and Photophysical Properties of a Hydrated Lanthanum Ion Complex with a Triazole Ligand by Several DFT Methods. Molecules.

[49] Lutoshkin M.A. (2025). Solvation Effects on the Sustainability of Lanthanum Complexes. J. Phys. Chem. A.

[50] Palafox M.A., Belskaya N.P., Todorov L.T., Hristova-Avakoumova N.G., Kostova I.P. (2024). Effect of Lanthanide Ions and Triazole Ligands on the Molecular Properties, Spectroscopy and Pharmacological Activity. Int. J. Mol. Sci..

[51] Kühn F.E., Kratzert D., Stalke D., Roesky P.W., Andruh M. (2024). Magnetic and spectroscopic properties of chloride-bridged guanidinate dilanthanide complexes. Polyhedron.

[52] Delano F., Demir S. (2024). Unprecedented Pyrazine-Bridged Guanidinate Rare Earth Complexes Through a Bridge Splitting Reaction Path. Eur. J. Inorg. Chem..

[53] Roesky P.W., Jenter J., Köppe R. (2010). Mixed borohydride-chloride complexes of the rare earth elements. Comptes Rendus Chim..

[54] Al Momani W.M., Taha Z.A., Ajlouni A.M., Shaqra Q.M.A., Al Zouby M. (2013). A study of in vitro antibacterial activity of lanthanides complexes with a tetradentate Schiff base ligand. Asian Pac. J. Trop. Biomed..

[55] Tipper D.J., Strominger J.L. (1965). Mechanism of action of penicillins: A proposal based on their structural similarity to acyl-D-alanyl-D-alanine. Proc. Natl. Acad. Sci. USA.

[56] Biegański P., Szczupak Ł., Arruebo M., Kowalski K. (2021). Brief Survey on Organometalated Antibacterial Drugs and Metal-Based Materials with Antibacterial Activity. RSC Chem. Biol..

[57] Aslam B., Wang W., Arshad M.I., Khurshid M., Muzammil S., Rasool M.H., Baloch Z. (2018). Antibiotic resistance: A rundown of a global crisis. Infect. Drug Resist..

[58] Moreno-Latorre M., de la Torre M.C., Cabeza J.A., García-Álvarez P., Sierra M.A. (2024). Attaching Metal-Containing Moieties to β-Lactam Antibiotics: The Case of Penicillin and Cephalosporin. Inorg. Chem..

[59] Valaperta R., Tejada M.R., Frigerio M., Moroni A., Ciulla E., Cioffi S., Capelli P., Costa E. (2010). *Staphylococcus aureus* nosocomial infections: The role of a rapid and low-cost characterization for the establishment of a surveillance system. New Microbiol..

[60] Bertoncheli C.D.M., Hörner R. (2008). Uma revisão sobre metalo-β-lactamases. Rev. Bras. Ciênc. Farm..

[61] Okeke I.N., de Kraker M.E., Van Boeckel T.P., Kumar C.K., Schmitt H., Gales A.C., Laxminarayan R. (2024). The scope of the antimicrobial resistance challenge. Lancet.

[62] Suffness M., Pezzuto J.M., Hostettmann K. (1990). Assays Related to Cancer Drug Discovery. Methods in Plant Biochemistry: Assays for Bioactivity.

[63] Monks A., Scudiero D., Skehan P., Shoemaker R., Paull K., Vistica D., Hose C., Langley J., Cronise P., Vaigro-Wolff A. (1991). Feasibility of a high-flux anticancer drug screen using a diverse panel of cultured human tumor cell lines. J. Natl. Cancer Inst..

[64] Boyd M.R., Paull K.D. (1995). Some practical considerations and applications of the National Cancer Institute in vitro anticancer drug discovery screen. Drug Dev. Res..

[65] Ferraz R., Costa-Rodrigues J., Fernandes M.H., Santos M.M., Marrucho I.M., Rebelo L.P.N., Branco L.C. (2015). Antitumor activity of ionic liquids based on ampicillin. ChemMedChem.

[66] Alexander C., Thom J.A., Kenwright A.M., Christensen K.E., Sørensen T.J., Faulkner S. (2023). Chelating chloride using binuclear lanthanide complexes in water. Chem. Sci..

[67] Nolin F., Michel J., Wortham L., Tchelidze P., Balossier G., Banchet V., Bobichon H., Lalun N., Terryn C., Ploton D. (2013). Changes to cellular water and element content induced by nucleolar stress: Investigation by a cryo-correlative nano-imaging approach. Cell. Mol. Life Sci..

[68] Finney A.R., Lectez S., Freeman C.L., Harding J.H., Stackhouse S. (2019). Ion Association in Lanthanide Chloride Solutions. Chemistry.

[69] Aramesh-Boroujeni Z., Jahani S., Khorasani-Motlagh M., Kerman K., Noroozifar M. (2020). Evaluation of parent and nano-encapsulated terbium(III) complex toward its photoluminescence properties, FS-DNA, BSA binding affinity, and biological applications. J. Trace Elem. Med. Biol..

[70] Singh R., Lillard J.W. (2009). Nanoparticle-based targeted drug delivery. Exp. Mol. Pathol..

[71] Torchilin V.P. (2005). Recent advances with liposomes as pharmaceutical carriers. Nat. Rev. Drug Discov..

[72] Peer D., Karp J.M., Hong S., Farokhzad O.C., Margalit R., Langer R. (2020). Nanocarriers as an emerging platform for cancer therapy. Nano-Enabled Medical Applications.

[73] Maeda H., Wu J., Sawa T., Matsumura Y., Hori K. (2000). Tumor vascular permeability and the EPR effect in macromolecular therapeutics: A review. J. Control Release.

[74] Jorgensen J.H., Turnidge J.D. (2015). Susceptibility test methods: Dilution and disk diffusion methods. Manual of Clinical Microbiology.

[75] Papazisis K.T., Geromichalos G.D., Dimitriadis K.A., Kortsaris A.H. (1997). Optimization of the sulforhodamine B colorimetric assay. J. Immunol. Methods.

[76] Frisch M.J., Trucks G.W., Schlegel H.B., Scuseria G.E., Robb M.A., Cheeseman J.R., Scalmani G., Barone V., Petersson G.A., Nakatsuji H. (2016). Gaussian 16, Revision A.03.

[77] Binning R.C., Curtiss L.A. (1990). Compact contracted basis-sets for 3rd-row atoms—Ga-Kr. J. Comp. Chem..

[78] Blaudeau J.-P., McGrath M.P., Curtiss L.A., Radom L. (1997). Extension of Gaussian-2 (G2) theory to molecules containing third-row atoms K and Ca. J. Chem. Phys..

[79] Rassolov V.A., Ratner M.A., Pople J.A., Redfern P.C., Curtiss L.A. (2001). 6-31G* Basis Set for Third-Row Atoms. J. Comp. Chem..

[80] Ditchfield R., Hehre W.J., Pople J.A. (1971). Self-Consistent Molecular Orbital Methods. 9. Extended Gaussian-type basis for molecular-orbital studies of organic molecules. J. Chem. Phys..

[81] Chemcraft—Graphical Software for Visualization of Quantum Chemistry Computations. https://www.chemcraftprog.com.

[82] Barge A., Cravotto G., Gianolio E., Fedeli F. (2006). How to determine free Gd and free ligand in solution of Gd chelates. A technical note. Contrast Media Mol. Imaging.

[83] El-Shenawy A.I., Atta A.H., Refat M.S. (2014). Complexation of Gadolinium (III) and Terbium (III) with nalidixic acid (NDX): Molar conductivity, thermal and spectral investigation. Int. J. Electrochem. Sci..

[84] Geary W.J. (1971). The use of conductivity measurements in organic solvents for the characterisation of coordination compounds. Coord. Chem. Rev..

