# Supplementary material for: Synthesis, Spectroscopy Characterization and Biological Evaluation of La(III), Eu(III) and Gd(III) Complexes with Ampicillin: In Vitro Antimicrobial, Cytotoxic and Antiproliferative Activities and Theoretical Frameworks
Source: Molecules. 2026 Apr 28;31(9):1465. doi: 10.3390/molecules31091465 (PMC13164664; doi:10.3390/molecules31091465)
Supplement: Supplementary file 1 [file molecules-31-01465-s001.zip › molecules-4235402-supplementary.pdf]

Supplementary Information

Synthesis, Spectroscopy Characterization and Biological Evaluation of La(III), Eu(III) and Gd(III) Complexes with Ampicillin: *in vitro* Antimicrobial, Cytotoxic and Antiproliferative activities and Theoretical Frameworks

Diego Boldo [1], Vasilii Khripun [1], Kristiane Fanti Del Pino [1], Juliana Jorge [1], Luana da Silva Oliveira [1], Danielle Bogo [2], Ana Camila Micheletti [1], Adriana Pereira Duarte [1], Hernane da Silva Barud [3], Ariadna Lafourcade Prada[2], Teofilo Fernando Mazon Cardoso[2]; Gustavo Rocha de Castro[4], Jesus Rafael Rodríguez Amado[5], Marco Antonio Utrera Martines [1,*]

[1] Chemistry Institute, Federal University of Mato Grosso do Sul, Campo Grande 79079-900, MS, Brazil; diego.boldo18@gmail.com (D.B.); vasilli.khripun@ufms.br (V.K.); kristianefdp@gmail.com (K.F.D.P.); juliana.jorge@ufms.br (J.J.); luana.s.oliveira@ufms.br (L.d.S.O.); anamicheletti@gmail.com (A.C.M.); adriana.duarte@ufms.br (A.P.D.)

[2] Faculty of Pharmacy, Food and Nutrition, Federal University of Mato Grosso do Sul, Campo Grande 79079-900, MS, Brazil; danielle.bogo@ufms.br (D.B.); ariadnalafu1977@gmail.com (A.L.P.); teofilo.cardoso@ufms.br (T.F.M.C.)

[3] Laboratory of Biopolymers and Biomaterials, University of Araraquara, Araraquara 14801-340, SP, Brazil; hernane.barud@gmail.com (H.d.S.B)

[4] Institute of Biosciences, São Paulo State University, Botucatu 18618-689, SP, Brazil; gustavo.castro@unesp.br (G.R.d.C)

[5] Faculty of Health Sciences, Federal University of Grande Dourados, Dourados CEP 79825-070, MS, Brazil; amado.jesus@ufam.edu.br (J.R.R.A)

* **Correspondence:** marco.martines@ufms.br

Supplementary Note 1 – Physical appearance of the synthesized complexes

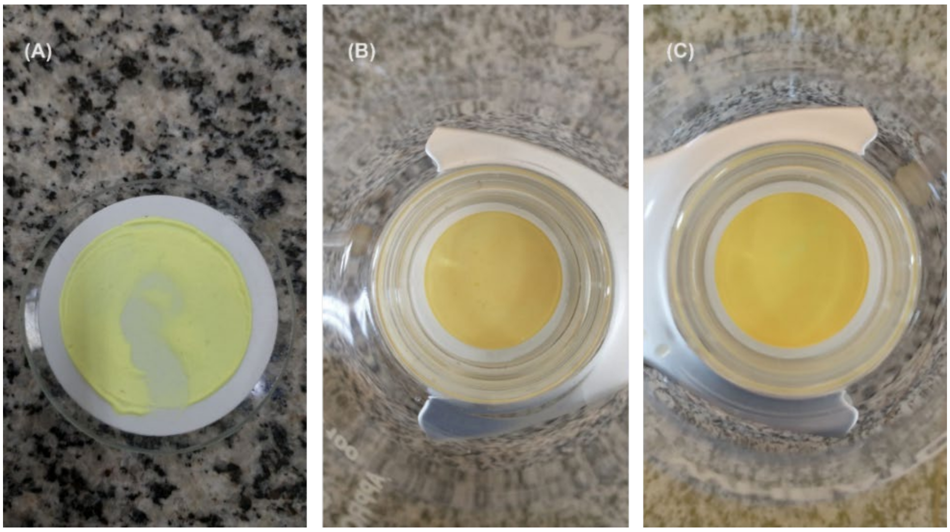

Figure S1. Physical appearance of the synthesized complexes with their respective colors: (A) Ampicillin coordinated with lanthanum (LaL); (B) Ampicillin coordinated with europium (EuL); (C) Ampicillin coordinated with gadolinium (GdL).

Supplementary Note 2 – DSC Analysis

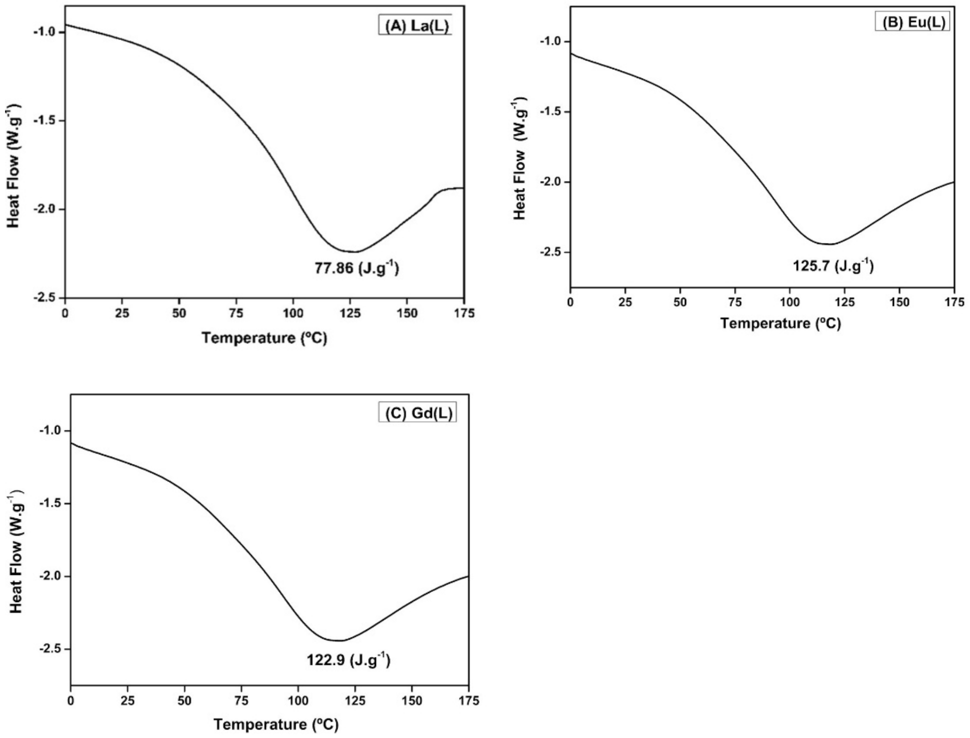

Figure S2. DSC analysis of complexes: (A) LaL = $[La_2(L)(Cl)_5(H_2O)_2]$, (B) EuL = $[Eu_2(L)(Cl)_5(H_2O)_5]$ and (C) GdL = $[Gd_2(L)(Cl)_5(H_2O)_5]$.

Table S1. Dehydration enthalpy values of the lanthanide complexes

	Enthalpy of dehydration (ΔH)	
**Samples**	**(J.g^{-1})**	**(Kj.mol^{-1})**
**[$La_2(L)(Cl)_5(H_2O)_2$]**	77.86	65.44
**[$Eu_2(L)(Cl)_5(H_2O)_5$]**	123.7	111.67
**[$Gd_2(L)(Cl)_5(H_2O)_5$]**	122.9	112.31

Supplementary Note 3 – Determination of lanthanide in the complexes by complexometric titration.

The complexometric titration analysis was used to determine the percentage of lanthanide present in the synthesized complexes, using Equation 1 [1]. For each analysis, an acid digestion process was performed with 20 µL of HCl P.A., the addition of 20 µL of deionized water, and 10 mg of each complex. The volumes of EDTA consumed during the titration are presented in Table S1, along with the determined percentage of lanthanide.

$$\%Ln^{3+} = \frac{MM_{(Ln^{3+})}.[EDTA].V_{EDTA}}{m_{(complex)}} x100 \qquad \textbf{(1)}$$

Table S2. Volumes of 0.01 mol·L^{-1} EDTA used in the complexometric titrations and the corresponding percentages of lanthanide in the respective samples.

Samples	Tritation 1 Volume (mL)	Tritation 2 Volume (mL)	Tritation 3 Volume (mL)	$V_{médio}$	% Ln
LaL	0.25	0.26	0.25	0.25	34.72
EuL	0.21	0.22	0.21	0.21	33.58
GdL	0.22	0.21	0.22	0.21	34.67

Supplementary Note 4 – X-ray Diffraction Analysis

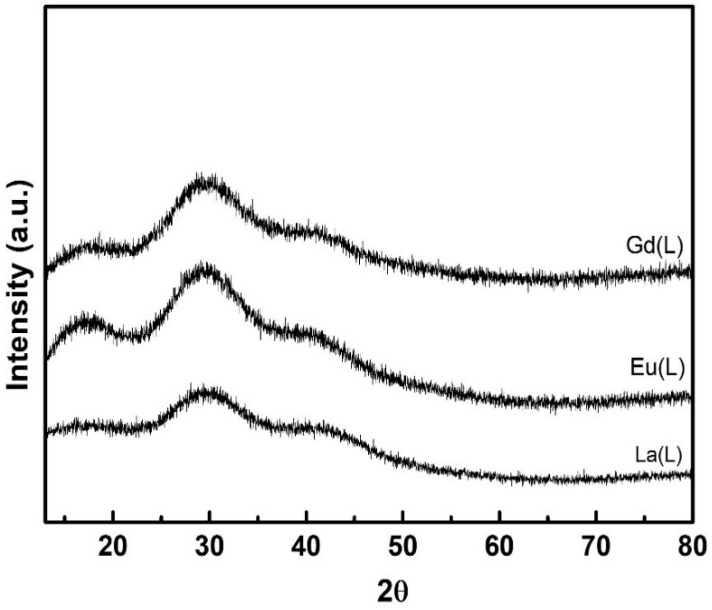

Figure S3. Diffractogram of the synthesized complexes, LaL, EuL, and GdL.

Supplementary Note 4 – Electronic spectra of the ligand and the synthesized complexes

Table S3. Assignments of the bands and their respective electronic transitions for the ligand (NaL) and the corresponding complexes $[La_2(L)(Cl)_5(H_2O)_2]$, $[Eu_2(L)(Cl)_5(H_2O)_5]$, and $[Gd_2(L)(Cl)_5(H_2O)_5]$, where L = sodium ampicillin salt.

	Transitions/ $\lambda_{máx}$ (nm)	
Samples	$\pi \rightarrow \pi^*$	$n \rightarrow \pi^*$
NaL	266	342; 377
$[La_2(L)(Cl)_5(H_2O)_2]$	267	342; 384
$[Eu_2(L)(Cl)_5(H_2O)_5]$	267	343; 381
$[Gd_2(L)(Cl)_5(H_2O)_5]$	267	342; 381

Supplementary Note 5 – ^{1}H NMR Spectroscopy

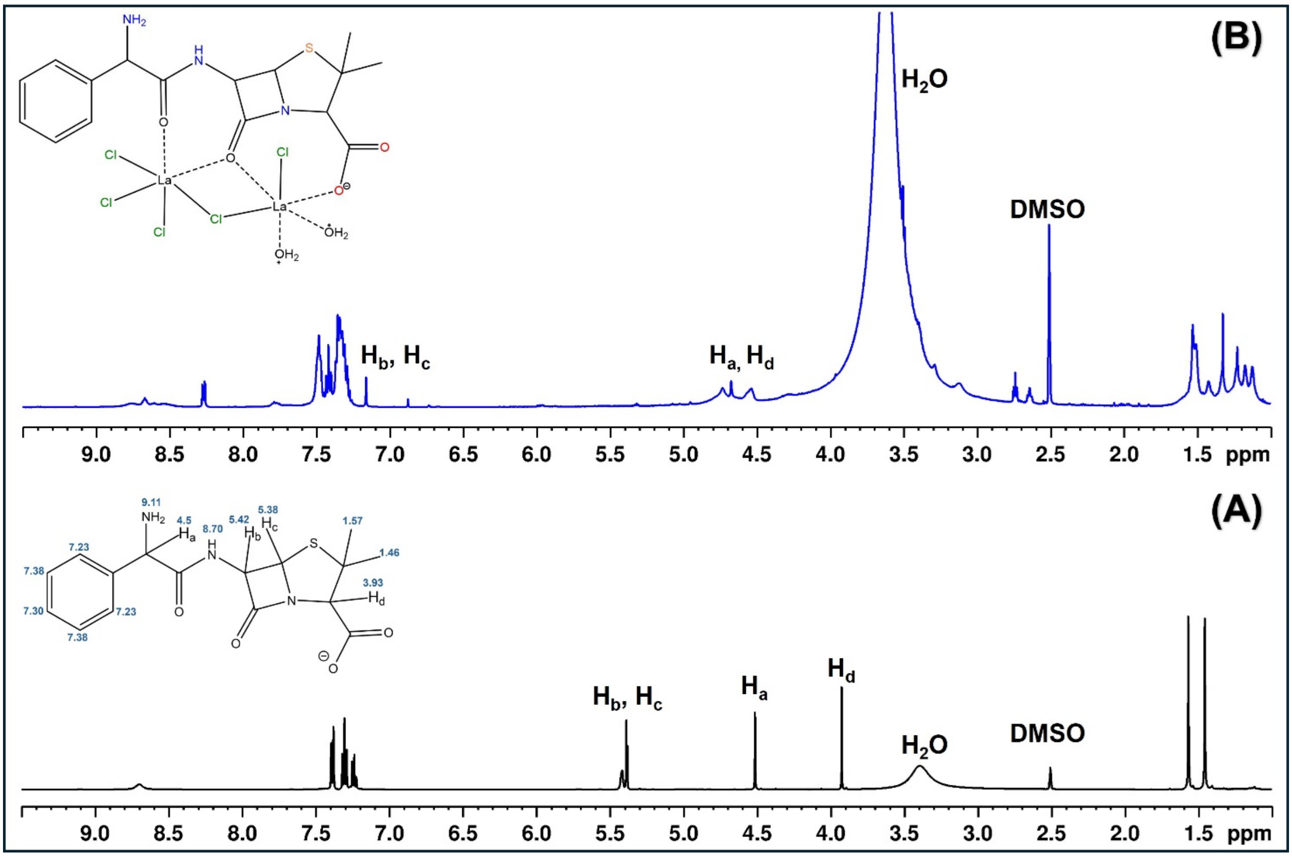

Figure S4. ^{1}H NMR spectra in DMSO-d_6: (A) of the ligand; (B) $[La_2(L)(Cl)_5(H_2O)_2]$

Supplementary Note 6 – Molar conductance for ligand and complexes

Table S4. Electrical conductivity measurements of the ligand and the synthesized complexes at a concentration of 10^{-3} M in DMSO at 26ºC at 0, 24, 48, and 72 hours. The data presented is the average of three readings taken for each sample.

	$\Lambda_m/\Omega m^{-1}\ cm^2\ mol^{-1}$			
Samples	0h	24h	48h	72h
NaL	25	24	24	23
$[La_2(L)(Cl)_5(H_2O)_2]$	30	29	28	28
$[Eu_2(L)(Cl)_5(H_2O)_5]$	29	28	28	27
$[Gd_2(L)(Cl)_5(H_2O)_5]$	30	29	28	28

The molar conductance data has a supporting item that has been used to speculate the conformational structure of metal chelates [2]. The measurements obtained in solution through analysis can help in the elucidation of coordination formulas, since high molar conductivity values indicate the proposal of electrolytic coordination complexes, and thus,

in the case of synthesized complexes, low conductivity indicating that both complexes are neutral, thus, with all chloride ions present in the inner coordination sphere [3]. Furthermore, the molar conductivity values in DMSO indicate that the synthesized complexes are stable, since the conductivity values do not change drastically within 72 h.

Supplementary Note 7 – DFT calculations

Table S5. Coordination of atoms in $La_2(L)(Cl)_5(H_2O)_2$ complex

**C**	**-4.916020000000**	**1.278230000000**	**-0.919695000000**
**C**	**-5.240603000000**	**0.194603000000**	**-1.736029000000**
**C**	**-5.645082000000**	**1.489900000000**	**0.254831000000**
**C**	**-6.272772000000**	**-0.670650000000**	**-1.376205000000**
**C**	**-6.672029000000**	**0.624328000000**	**0.616528000000**
**C**	**-6.987841000000**	**-0.460063000000**	**-0.200518000000**
**C**	**-3.799849000000**	**2.248719000000**	**-1.269972000000**
**C**	**-2.531159000000**	**2.018039000000**	**-0.452236000000**
**N**	**-3.372909000000**	**2.343725000000**	**-2.665466000000**
**O**	**-2.485368000000**	**1.309487000000**	**0.598406000000**
**N**	**-1.438607000000**	**2.649278000000**	**-0.896681000000**
**C**	**-0.195327000000**	**2.599187000000**	**-0.181448000000**
**C**	**0.795773000000**	**1.435435000000**	**-0.429701000000**
**C**	**0.953387000000**	**3.510737000000**	**-0.690135000000**
**N**	**1.775532000000**	**2.296239000000**	**-0.827771000000**
**O**	**0.743590000000**	**0.200007000000**	**-0.256038000000**
**S**	**1.922804000000**	**4.633252000000**	**0.458128000000**
**C**	**3.369836000000**	**3.340140000000**	**0.640285000000**
**C**	**3.212701000000**	**2.458559000000**	**-0.635715000000**
**C**	**3.162667000000**	**2.559563000000**	**1.936834000000**
**C**	**4.692666000000**	**4.097266000000**	**0.637155000000**
**C**	**3.928812000000**	**1.118516000000**	**-0.615714000000**
**O**	**3.351903000000**	**0.178483000000**	**-1.302535000000**
**O**	**4.997656000000**	**0.966532000000**	**0.029189000000**
**La**	**-1.715244000000**	**-0.983628000000**	**0.820998000000**
**H**	**-1.529474000000**	**3.031794000000**	**-1.837103000000**
**Cl**	**-3.779589000000**	**-2.218857000000**	**1.871795000000**
**Cl**	**-0.059445000000**	**-0.022204000000**	**2.878916000000**
**Cl**	**-1.827594000000**	**-0.746248000000**	**-1.935140000000**
**La**	**2.454944000000**	**-1.818035000000**	**-0.532514000000**
**Cl**	**0.145462000000**	**-3.121086000000**	**0.427510000000**
**Cl**	**3.733466000000**	**-3.746344000000**	**-1.857021000000**
**O**	**2.571684000000**	**-1.075221000000**	**1.781625000000**
**O**	**4.797956000000**	**-1.661391000000**	**0.375100000000**
**H**	**5.395902000000**	**-2.324625000000**	**-0.013378000000**
**H**	**5.136259000000**	**-0.720203000000**	**0.273456000000**
**H**	**1.774686000000**	**-0.772547000000**	**2.301935000000**
**H**	**3.375435000000**	**-1.205857000000**	**2.310593000000**
**H**	**-4.680178000000**	**0.006480000000**	**-2.642699000000**

H	-5.399767000000	2.330422000000	0.897159000000
H	-6.507382000000	-1.516660000000	-2.011450000000
H	-7.220216000000	0.791149000000	1.536251000000
H	-7.781089000000	-1.141459000000	0.083305000000
H	-4.139050000000	3.252060000000	-0.974367000000
H	-3.031724000000	1.461698000000	-3.039754000000
H	-4.041024000000	2.795027000000	-3.276191000000
H	-0.372469000000	2.691419000000	0.893846000000
H	0.752572000000	4.028591000000	-1.627107000000
H	3.597604000000	3.021422000000	-1.494966000000
H	3.125123000000	3.248958000000	2.782808000000
H	3.998436000000	1.871131000000	2.084358000000
H	2.231035000000	1.986048000000	1.944687000000
H	4.811476000000	4.694884000000	-0.270143000000
H	4.757959000000	4.766779000000	1.498346000000
H	5.510555000000	3.373862000000	0.697639000000

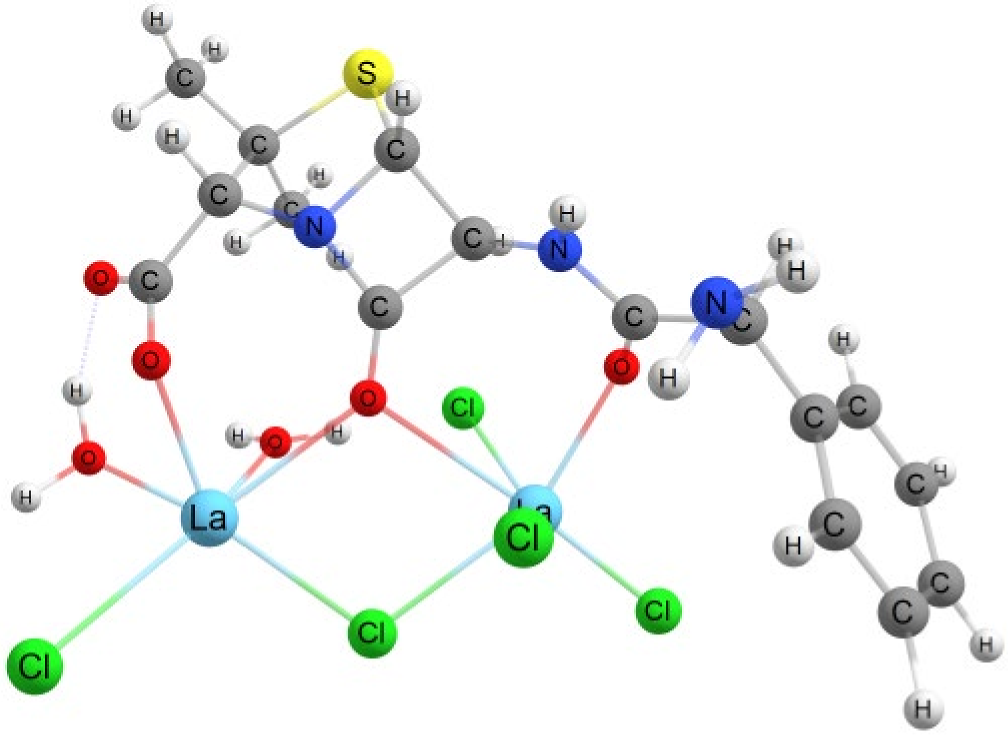

Figure S5. The structure of calculated $La_2(L)(Cl)_5(H_2O)_2$ complex

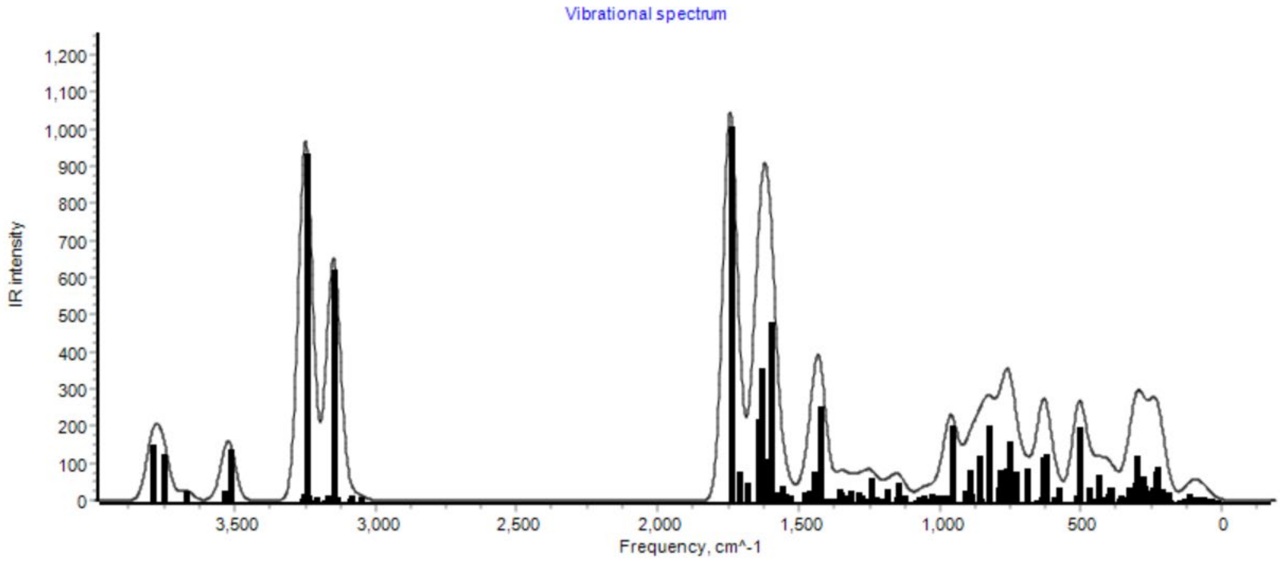

Figure S6. Calculated IR spectrum of the $La_2(L)(Cl)_5(H_2O)_2$ complex

REFERENCES

1. Barge, Alessandro, et al. How to determine free Gd and free ligand in solution of Gd chelates. A technical note. *CMMI* 1.5 (2006): 184-188. **https://doi.org/10.1002/cmmi.110**
2. El-Shenawy, Ahmed I., Aly H. Atta, and Moamen S. Refat. Complexation of Gadolinium (III) and terbium (III) with nalidixic acid (NDX): Molar conductivity, thermal and spectral investigation. *Int. J. Electrochem. Sci* 9.9 (2014): 5187-5203. https://doi.org/10.1016/S1452-3981(23)08160-9
3. Geary, William J. The use of conductivity measurements in organic solvents for the characterisation of coordination compounds. *Coord. Chem. Rev.* 7.1 (1971): 81-122. https://doi.org/10.1016/S0010-8545(00)80009-0
